# Effects of Feed Solution pH on Polyelectrolyte Multilayer Nanofiltration Membranes

**DOI:** 10.1021/acsapm.2c01542

**Published:** 2022-12-20

**Authors:** Moritz A. Junker, Jurjen A. Regenspurg, Cristobal I. Valdes Rivera, Esra te Brinke, Wiebe M. de Vos

**Affiliations:** † Membrane Science and Technology, University of Twente, MESA+ Institute for Nanotechnology, P.O. Box 217, 7500 AEEnschede, The Netherlands; ‡ International Institute for Infrastructural, Hydraulic and Environmental Engineering—IHE, P.O. Box 3015, 2601 DADelft, The Netherlands

**Keywords:** polyelectrolyte multilayer, nanofiltration, membranes, pH sensitivity, hollow fiber

## Abstract

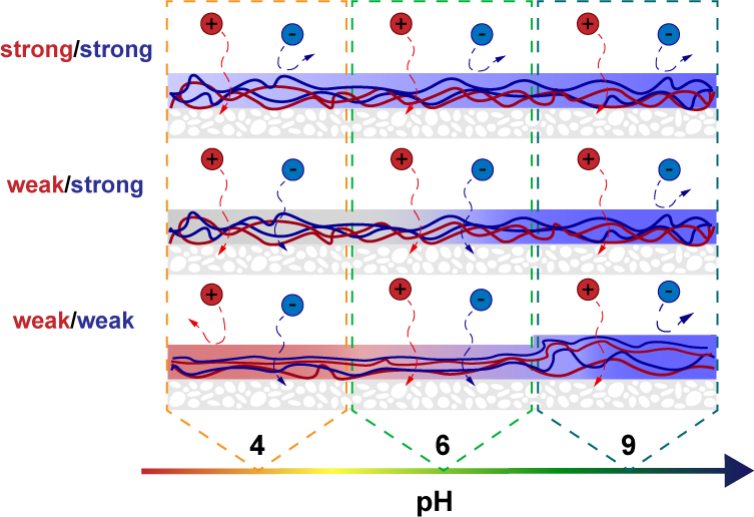

Over the past decade polyelectrolyte multilayer (PEM)-based membranes have gained a lot of interest in the field of nanofiltration (NF) as an alternative to conventional polyamide-based thin film composite membranes. With great variety in fabrication conditions, these membranes can achieve superior properties such as high chemical resistance and excellent filtration performance. Some of the most common polyelectrolytes used to prepare NF membranes are weak, meaning that their charge density depends on pH within the normal window of operation relevant for potential applications (pH 0–14). This might cause a dependency of membrane properties on the pH of filtered solutions, as indicated by other applications of PEMs. In this work, the susceptibility of membrane structure (swelling and surface charge) and performance (permeability, molecular weight cutoff, and salt retention) toward the pH of the filtration solution was studied for four fundamentally different PEM systems: poly(diallyldimethylammonium chloride) (PDADMAC)/poly(sodium-4-styrenesulfonate) (PSS) (strong/strong), poly(allylamine hydrochloric acid) (PAH)/poly(acrylic acid) (PAA) (weak/weak), and PAH/PSS (weak/strong) and PAH/PSS+PAH/PAA (asymmetric). Slight variations in structure and performance of the PDADMAC/PSS-based membranes were observed. On the contrary, structure and performance of PAH/PAA-based membranes are very susceptible to feed solution pH. A continuous change in charge density with variation in pH significantly affects salt retention. An increased swelling at pH 9 translates to variation in permeability and molecular weight cutoff of the membrane. The susceptibility of PAH/PSS-based membranes to pH is less pronounced compared to the PAH/PAA-based membranes since only one of the polyelectrolytes involved is weak. No structural changes were observed, indicating additional specific interactions between the polyelectrolytes other than electrostatic forces that stabilize film structure. A combination of the PAH/PSS and PAH/PAA system (8 + 2 bilayers) also displays a clear dependency of both membrane structure and performance on solution pH, where PAH/PSS is dominating due to a higher bilayer number.

## Introduction

In recent years nanofiltration (NF) type membranes have shown great potential in numerous fields and applications such as hardness removal,^1^ organic micropollutant removal,^2^ and nitrate removal.^3^ With pore sizes on the order of magnitude of 1 nm, molecular weight cutoff (MWCO) values between 200 and 1000 Da, and sodium chloride retention values of 20–80%, NF membranes have properties which fall in between ultrafiltration (UF) and reverse osmosis (RO) type membranes.^4^ These typical values are obtained at relatively high fluxes which, compared to RO type membranes, are achieved at lower operating pressures and hence lower operating costs. Besides rejecting solutes based on size, NF membranes combine the size exclusion effect with separations based on the affinity between solutes and membrane.^5^ Additionally, in the case of charged solutes, electrostatic interactions between the membrane and solutes can strongly influence separation properties. The most common NF membranes are polyamide-based thin-film composite membranes. By means of interfacial polymerization, a thin selective film is fabricated on top of a porous support membrane resulting in RO and NF type membranes,^6^ a process which is well established for flat sheet membranes but is more difficult to perform for membranes with a hollow fiber (HF) geometry.^7^

A very promising and less challenging alternative to polyamide-based HF NF membranes is the use of polyelectrolyte multilayers (PEMs). Over the last years, PEMs have found their way into a broad range of applications, among others, optics^8^ and drug delivery.^9^ Also in the field of membrane science PEMs have been shown to have great potential.^10,11^ First introduced by Decher et al. in 1992,^12^ it was shown that exposing a charged substrate to polycations and polyanions in an alternating fashion results in the self-assembly of a multilayered thin film due to the gain in entropy by the release of counterions upon layer formation. By employing the layer-by-layer (LbL) technique,^13^ PEMs can be fabricated onto a HF support membrane. For this, a charged UF HF membrane is alternatingly exposed to polycations and polyanions resulting in a thin PEM which allows for increased selectivity.

By careful choice of polyelectrolytes (PEs) and fabrication conditions, great control over PEM fabrication and thus final properties is obtained. The tuneability of PEMs allows for targeting specific applications, e.g., water softening and desalination^14^ or the removal of organic micropollutants.^15^ Bruening and co-workers have shown that it is possible to have selective ion rejections using poly(diallyldimethylammonium chloride) (PDADMAC)/poly(sodium-4-styrenesulfonate) (PSS) multilayer NF membranes, reporting phosphate rejection of 86–98% (depending on pH) with a chloride/phosphate selectivity of 48.^16^ Similarly, Wang et al. have used PDADMAC/PSS to fabricate NF membranes with, compared to commercial NF membranes, similarly high retention toward organic micropollutants (around 90%), slightly lower pure water permeability, but at the same time low retention toward divalent cations (below 20%).^17^ These properties result in a lower scaling potential compared to commercial NF membranes. In a recent study by Elshof et al.,^18^ it was shown that the filtration performance of NF membranes based on the frequently applied PEs PDADMAC and PSS remained stable after more than two months of exposure to basic (pH 14) and acidic (pH 0) conditions. This outstanding pH stability allows for potential applications of these membranes under harsh conditions found in industrial processes such as treatment of mining effluent,^19^ treatment of dairy cleaning solutions,^20^ sulfate removal in vacuum salt production,^21^ and hemicellulose removal in viscous fiber production.^22^ The high chemical resistance of these membranes^23^ also allows the use of common cleaning methods to mitigate membrane fouling.

One way to exploit the versatility of the LbL method was recently displayed by te Brinke et al.,^15^ where the concept of an asymmetric structure within the PEM layer was utilized to fabricate high-performance NF membranes for micropollutant removal. These membranes were based on PSS/poly(allylamine hydrochloric acid) (PAH) (as a more open support layer) and poly(acrylic acid) (PAA)/PAH (as a dense separation layer).

Even though the influence of pH (during coating^24−28^ and postassembly^29−45^) on PEM properties and (postassembly) on the stability of PEM NF membranes^18^ have been widely studied, the influence of pH (within the range of stability) on the performance on NF PEM membranes has to the best of our knowledge not been studied, yet.

The potential influence of pH on ion transport through PEM films was already illustrated by Ahmad et al., who used PAH/PSS coatings on top of ion exchange membranes to increase selectivity. Here, already small variations in solution pH (from pH 6.5 to 8.3) significantly altered ion transport (and likely excess charge of the PEM film).^46^

The expected influence of pH on PEM NF membranes based on current knowledge can be divided into three categories that will be summarized in the following.

First, for conventional polyamide-based membranes used in NF processes the influence of solution pH on filtration performance has already been studied intensively.^47−53^ Here pH can affect filtration performance by influencing both membrane properties and solution properties. Second, the influence of solution pH during LbL coating on final PEM properties (with at least one weak PE involved) has been shown in multiple studies,^24−27^ also for the application of NF.^28^ The dissociation behavior of functional groups attributing to the overall charge density depends on solution pH. Here we can distinguish between weak (e.g., PAH and PAA) and strong (e.g., PDADMAC and PSS) PEs. Strong PEs are characterized by a charge density that is independent of solution pH (here defined for a range of pH 0–14). Weak PEs, on the other hand, exhibit a dependency of charge density as a function of pH within this range. The group of Rubner has contributed a lot to clarifying the influence of PE charge density on PEM formation.^25−27^ The charge density of weak PEs (controlled via pH) influences the conformation in which they are incorporated into the multilayer. As a result, properties such as layer thickness, composition, surface wettability, and layer interpenetration can be controlled. Third, the responsiveness of PEMs (postassembly) toward external stimuli, such as pH, has been shown in multiple studies.^29−45^ These properties are especially interesting for loading and releasing of target molecules, either within a capsule^29,36,37^ or the PEM^30,31^ itself, with potential applications in the biomedical field. Here, the buildup of excess charge as a result of pH changes can cause swelling, changes in film morphology, or even break down PEMs.^41^ To prevent the PEM from falling apart, attractive forces (hydrogen bonding, hydrophobic interactions, electrostatic attraction) must outweigh the repulsive forces (electrostatic repulsion, osmotic pressure).^33,34,37,39,41^

Thus, solution pH might have a severe influence on the membrane performance under certain conditions and has to be considered in NF applications. In addition, investigating the behavior of PEM membranes under different pH conditions might offer a different perspective compared to conventional characterization methods and improve our understanding of the fundamental mechanisms of PEMs. In this work, we systematically study the influence of feed solution pH on the performance of PEM NF membranes. The PEM systems under investigation are frequently used for the fabrication of NF membranes and cover the three fundamentally different configurations weak/weak (PAH/PAA), weak/strong (PAH/PSS), and strong/strong (PDADMAC/PSS). Through a combination of membrane performance measurements with swelling and surface charge measurements, intrinsic PEM properties are inferred. Lastly, we extend our investigation to asymmetric PEM membranes developed by te Brinke et al. (PAH/PSS + PAH/PAA).^15^

## Experimental Section

The sensitivity of PEM NF membranes toward solution pH was investigated by experimentally studying these films under slightly acidic (pH 4), neutral (pH 6), and slightly basic (pH 9) conditions. Moderate variations in pH were chosen to ensure the stability of the PEM membranes under repeated and long-term operation but also to remain in the pH window relevant to typical water treatment applications. Also, it was shown previously that already small changes in pH (from pH 6.5 to 8.3) can significantly affect ion transport through PEM layers as ion exchange membranes.^46^ Therefore, we did expect the filtration performance of the chosen PEM NF membranes to be sensitive to pH within the chosen range. Lastly, we expect any effects on PEM structure within this moderate pH range to be intensified when going to more extreme pH values.

### Materials

Modified poly(ethersulfone) HF UF membranes were kindly provided by NX Filtration B.V., Enschede, The Netherlands. The provided membranes have an approximate 90% MWCO of 10 kDa and an inner diameter of 0.7 mm. Poly(allylamine hydrochloric acid) (PAH, *M*_w_ = 150.000 g mol^–1^, 40 wt % in water) was obtained from Nittobo medical CO., LTD, Japan. Poly(acrylic acid) (PAA, *M*_w_ = 250.000 g mol^–1^, 35 wt % in water), poly(sodium-4-styrenesulfonate) (PSS, *M*_w_ = 200.000 g mol^–1^, 30 wt % in water), poly(diallyldimethylammonium chloride) (PDADMAC, *M*_w_ = 200.000–350.000 g mol^–1^, 20 wt % in water), glycerol solution (86–89%), MgCl_2_ hexahydrate (purity 99%), sodium hydroxide (pellets, purity 98%), and hydrchloric acid (ACS reagent, 37%) were purchased from Sigma-Aldrich. H_2_SO_4_ (96% in H_2_O) was purchased from Acros Organics B.V.B.A. NaCl (purity 99.9 wt %) was kindly provided by Nouryon Industrial Chemicals. H_2_O_2_ (30 w/w% solution in H_2_O), Na_2_SO_4_ decahydrate, ethylene glycol, and polyethylene glycol (PEG) of various *M*_w_ (200, 400, 600, and 1000 Da) were obtained from Merck. Diethylene glycol was purchased from Sigma-Aldrich. All chemicals were used without any purification.

### Methods

Pure water permeability, salt retention, and MWCO measurements have been performed in a crossflow-mode at a transmembrane pressure of 5 bar, 20 ± 0.5 °C, and a cross-flow velocity of 1 ms^–1^. A schematic of the cross-flow setup that was used can be found in Figure S1.

#### Membrane Fabrication

The obtained HF UF membranes were modified with multiple combinations of the 4 types of PEs. Modification was done by dipcoating based on the LbL principle. The HF UF membranes were alternately exposed to polycation and polyanion, starting with the polycation because of the negative surface charge of the support membranes. Both aqueous PE solutions were set at pH 5.5 and an ionic strength of 50 mM NaCl. The HFs were immersed in the PE solutions for 15 min. In between PE solutions, the HFs were rinsed using aqueous solutions of 50 mM NaCl, to remove loosely bound PEs without influencing the salt concentration of the PEM. The rinsing step was performed 3 times, for 5 min each. One bilayer was obtained when the HFs had been exposed to polycation and polyanion, with 3 rinsing steps in between. Depending on the combination of polycation and polyanion, a specific amount of bilayers was coated onto the HF support membranes. Dipcoating resulted in PEM membranes of the following composition (depicted as [polycation/polyanion]_#BL_); symmetric [PDADMAC/PSS]_6/6.5_, [PAH/PAA]_8/8.5_, [PAH/PSS]_8/8.5_, and asymmetric [PAH/PSS]_8_+[PAH/PAA]_2/2.5_. It should be mentioned that even though the performed dip coating leads to layer deposition on both the inside and outside of the HF UF membrane, an asymmetric pore structure with the dense layer being located on the inside of the HF results in defect-free layer formation only on the inside of the HF.

After dipcoating, the HFs were placed into an aqueous solution of 15% glycerol for 4 h. Consequently, the HFs were taken out and hung to dry overnight. Subsequently, modules were fabricated by placing the HFs into plastic tubing with an outer diameter of 6 mm and a length of approximately 26 cm. Both outer ends (4 cm) of the tubing were potted using a 2 component polyurethane glue. Since the HFs were operated in an inside-out flow configuration, a hole was cut at the midpoint of the tubing enabling one to collect permeate. An example of a membrane module can be found in Figure S2.

#### Permeability

Pure water permeability had been obtained for all membranes which were made as mentioned under section Membrane Fabrication. The feed, for the measurements at pH 6, was Milli-Q water with a pH of approximately 5.8.
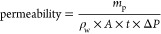
1

The permeate was collected and weighed, and with the use of eq 1, the permeability (L m^2–^ h^–1^ bar^–1^) was calculated for all samples. Where *m*_p_ is the permeate mass in g, ρ_w_ equals the density of water (1000 g L^–1^ at 20 °C), *A* is the active membrane area in m^2^, *t* is the duration of the experiment in h, and Δ*P* is the transmembrane pressure in bar.

The permeabilities at pHs 4 and 9 were obtained together with salt retention measurements. The reason for this is the ability to maintain a more stable pH value in salt solutions. Similar to the permeability measurements at pH 6, the permeate was weighed. Due to the salt concentration of 5 mM, it was necessary to compensate for osmotic pressure in calculations for permeability. Eq 2 was used to calculate the osmotic pressure difference.
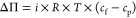
2

In eq 2 ΔΠ is the osmotic pressure difference across the membrane in Pa, i is the van ’t Hoff coefficient, R is the ideal gas constant (8.314 m^3^PaK^–1^ mol^–1^), T is temperature in Kelvin, c_f_ the salt concentration in the feed and c_p_ the salt concentration in the permeate in molm^–3^. The value for osmotic pressure, calculated using eq 2, is subtracted from ΔP in eq 1. The van ’t Hoff coefficient for NaCl was set to 2. For Na_2_SO_4_ and MgCl_2_ a van ’t Hoff coefficient of 3 was used.

#### Salt Retention

Salt retention experiments have been performed at pHs 4, 6, and 9 for NaCl, MgCl_2_, and Na_2_SO_4_. Feed solutions of the desired salt were prepared using Milli-Q water and a salt concentration of 5 mM. The pH of the feed solutions was set using 1 M solutions of either HCl or NaOH, and pH values were measured using a FiveEasy benchtop pH meter from Mettler-Toledo B.V.

Retention was determined by means of conductivity using a WTW ProfiLine portable conductivity meter. The conductivity of the feed solution was measured at the start and end of the experiments. The permeate conductivity was measured once at the end of the experiment. With the use of eq 3, the retention in % was calculated where *c*_p_ is the conductivity of the permeate and *c*_f_ is the averaged conductivity of the feed solution.
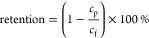
3

#### Molecular Weight Cut-Off

To study the relative change in pore size, MWCO measurements were performed. Again, these measurements were done at pHs 4, 6, and 9. A mixture of ethylene glycol, diethylene glycol, and PEGs varying in size from 200–1000 Da was used at a concentration of 1 g L^–1^ for each PEG. Permeate and feed samples were collected and analyzed using gel permeation chromatography (GPC, Agilent 1200/1260 Infinity GPC/SEC series). The GPC was set up with two columns in series obtained from Polymer Standards Service GmbH. The columns: Suprema 8 × 300 mm-1000 Å, 10 μm followed by 30 Å, 10 μm were operated at a flow of 1 mL min^–1^. Columns were followed by a refractive index detector. The refractive index variation due to PEG depends linearly on concentration and is therefore directly translated into retention. A feed sample was collected at the start and end of every experiment to determine the average signal intensity. From the obtained signal intensities the MWCO, defined as the molecular weight of PEG with 90% retention, was determined. Although the likelihood of the MWCO exactly matching the molecular weight of one of the PEG in solution is low, the overlap of peaks (normally distributed) leads to a continuous dependency of retention as a function of molecular weight (effectively interpolating). To illustrate this, a typical signal of a feed and permeate sample as well as the resulting sieving curve is displayed in Figure S3.

#### Zeta Potential

To obtain the surface charge of the various polyelectrolyte multilayer membranes (PEMMs), zeta potential measurements have been performed using the as prepared membranes. For this, an electrokinetic analyzer for solid surfaces (SurPASS, Anton Paar) was used. Sample modules have been prepared using tubing with an outer diameter of 8 mm to allow the placement of the HF PEMMs into the apparatus. Approximately 6–8 cm long pieces of single HF PEMMs were placed into this tubing and glued in place with a two-component polyurethane glue. The entire tubing around the HF PEMMs (shell side) was filled with glue such that there was only flow through the HF PEMMs without permeation. Measurements have been performed in pH titration mode in the range of pH 4 until pH 9 with an increment of 0.3 using a 5 mM KCl solution. Adjustments of pH were done using a 0.1 M solution of NaOH and HCl. During the measurements, the electrolyte solution containing 5 mM KCl is forced through the HF. Upon the flow of electrolyte through the HF, charge separation takes place at the HF inner surface. This charge separation leads to electrokinetic effects, among which the streaming potential is measured by the electrokinetic analyzer. Using the streaming potential and eq 4, the zeta potential, ζ (V), is calculated.
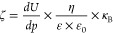
4

Here, *U* is the streaming potential in V, *p* is the applied pressure in Pa, η is the electrolyte viscosity in Pa s, ε is the dielectric coefficient of the electrolyte, ε_0_ is the permittivity of vacuum in A^2^s^4^kg^–1^m^–3^, κ_B_ is the electrolyte’s specific electrical conductivity in A^2^ s^3^ kg^–1^ m^–3^.

#### Ellipsometry

PEM swelling under different pH conditions was investigated by means of ellipsometry using a rotating compensator ellipsometer (Mk-2000 V and Mk-2000 X, J.A. Woollam Co., Inc.). Multilayers were coated on model surfaces, in our case silicon wafers, under the same conditions as the membranes (mentioned earlier). To ensure the silicon wafers were properly cleaned before coating, the wafers were treated with piranha solution (volumetric ratio H_2_SO_4_ (30  w/w%):H_2_O_2_ of 2:1) for 1 h after which they were treated with oxygen plasma for 15 min. First, the dry thickness was measured in N_2_ atmosphere. Wafers were mounted in a cell and exposed to a N_2_ flow of 400 mL min^–1^ for at least 30 min or until a stable signal was obtained (Mk-2000 X). Subsequently, the wet thickness was measured using a heated liquid cell with a volume of 5 mL (Mk-2000 V). Both dry and wet measurements were performed under an angle of 75° and a wavelength range of 380–1000 nm under ambient conditions. In the heated liquid cell, the wafers were exposed to Milli-Q and salt solutions of 5 mM NaCl with pHs 4, 6, and 9. After being exposed to Milli-Q, the wet cell was filled with the salt solution at different pH values. This was done in the following order: pHs 6–4–6–9–6.

For ellipsometry measurements, Si wafers with thermal SiO_2_ without doping were used. Optical constants of Si and SiO_2_ were taken from the software database (Complete Ease). The SiO_2_ thickness was determined to be 83 nm prior to film deposition. Layer thickness and refractive index of the PEM were fitted using a standard Cauchy model, shown in eq 5. Here, *n* is the refractive index of the layer, A and B are the Cauchy coefficients, and λ is the wavelength.
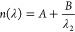
5In the Cauchy model, we assume that the PEM is a single layer.

Both the A and B parameters were determined from dry thickness measurements and kept constant in the following. For wet thickness measurements, the model was extended using a Bruggeman effective medium approximation (BEMA).
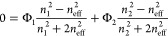
6

Here Φ is the volume fraction of the respective medium, *n* is the refractive index, and the indices indicate the two phases (“1”, “2”) and the medium representing the mixture of them (“eff”).

The swelling ratio (SR) of the PEMs on model surfaces was calculated using eq 7. Here, *d*_wet_ is the wet thickness of the PEM obtained under the various pH conditions, and *d*_dry_ is the PEM thickness obtained under N_2_ atmosphere.
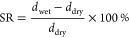
7

## Results and Discussion

The applied characterization methods give insight into the structural properties of PEMs under different solution pH conditions. We divide our discussion of results into two major parts: indirect characterization of the selective PEM layer (selective layer not applied in a filtration process) and direct characterization of the membrane performance (selective layer is applied during filtration). These two approaches can be seen as complementary techniques to study thickness, mesh size, and charge of the PEM systems.

### Indirect Characterization

#### Thickness and Swelling

Ellipsometry is a powerful tool to determine the layer thickness of thin films and subsequently swelling of these thin films.^54^ In this study, we have determined the dry layer thickness of the 4 PEM systems under a humid-free N_2_ atmosphere, to prevent any swelling due to humidity. To allow direct comparison of the systems and at the same time ensure a minimum film thickness of at least 10 nm, a total of 10 bilayers of each system (8 + 2 for asymmetric) was chosen. It is known that the sensitivity of ellipsometry toward external influences (such as angle offsets of the measurement cell) can deteriorate measurement accuracy at very low film thicknesses.^55^ In Table 1, the obtained dry thicknesses are shown for all PEM systems. Here the thickness of the PAH/PAA film stands out with a thickness of approximately 140 nm, whereas the other systems have thicknesses between 10 and 20 nm. Especially for PAH/PAA films, it is known that the bilayer thickness is dependent on the pH during coating. The obtained thickness for the PAH/PAA film is in good agreement with known values in literature^26^ and results from exponential layer growth.^56^

**Table 1:** Multilayer Thickness of 10 Bilayers under N_2_ Atmosphere

PEM system	thickness (nm)
PDADMAC/PSS	19.2 ± 0.1
PAH/PSS	14.3 ± 0.1
PAH/PAA	134 ± 4
asymmetric	13.5 ± 0.1

**Figure 1:**
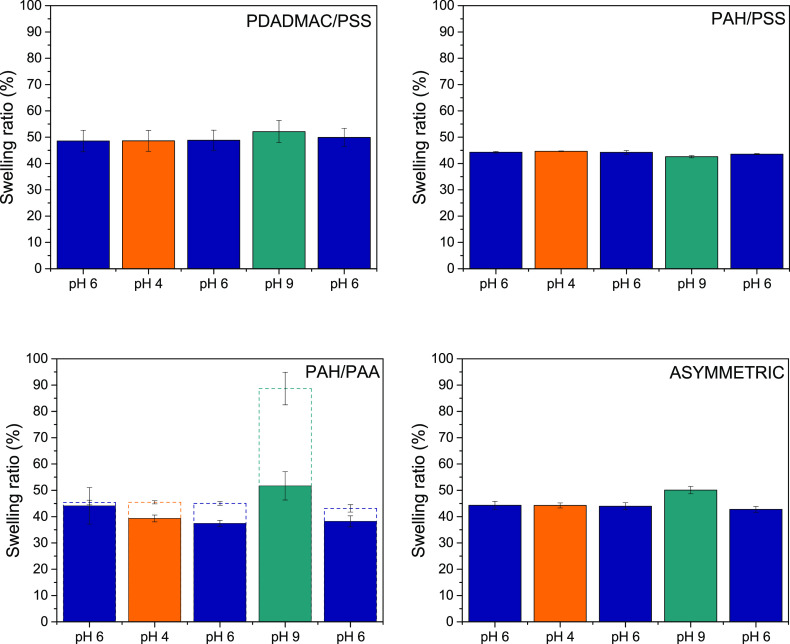
Swelling ratio (%) of negative ending PEM films on a silicon wafer determined via ellipsometry as a function of pH (−). The swelling ratio quantifies the increase in thickness of the PEM film (see eq 7) when exposed to 5 mM NaCl solution. Measurements at different pHs were performed in the displayed order (6–4–6–9–6). Three cycles (pHs 6–4–6–9–6) have been performed for the PAH/PAA multilayers. Data of the 1st cycle is indicated by dashed lines; the 3rd cycle is indicated by solid bars. Error bars display the standard error (sample size *n* = 3).

After dry film thickness determination, wet thicknesses under various pH conditions were measured. With the use of eq 7, the swelling ratio (%) as a function of pH was calculated, which is shown in Figure 1. It does need to be mentioned that the swelling ratio is obtained from PEM films deposited onto a model surface, meaning that the swelling ratio for PEM films deposited onto HF membranes could deviate. As expected for a strong/strong PEM system, there is no significant variation in swelling for PDADMAC/PSS within the pH range.

For PAH/PSS films, no change in swelling ratio was observed as a function of pH, although this would have been expected at pH 9 due to the deionization of PAH (p*K*_a_ between 8 and 9)^25^ and the resulting excess of negative charge.

In contrast to PAH/PSS films, the PAH/PAA films show a clear influence of pH on the swelling ratio at pH 9. Similar results with regard to the swelling ratio have been obtained by Tanchak et al. for PAH/PAA films fabricated at pH 5.^57^ The observed swelling at pH 9 can be explained by the presence of fully ionized PAA in combination with partially ionized PAH, at that pH. This would cause fewer ionic cross-links to be present, in turn causing more repulsion between the COO^–^ groups of PAA resulting in more swelling of the PAH/PAA film. In a similar fashion, one would expect a large increase in swelling around pH 4, since the p*K*_a_ value of PAA (in solution) is around 6.5.^25^

One explanation for the lack of swelling for the PAH/PSS system at pH 9 and the PAH/PAA system at pH 4 is a shift in effective p*K*_a_ when PEs are incorporated in multilayers, a phenomenon that has previously been observed.^58^ An additional explanation is the fact that the stability of PEMs is not solely determined by electrostatic interactions but rather by a balance of the sum of attractive and repulsive forces, which are specific for each PE combination.

It also needs mentioning that the multilayer seems to undergo reorganization during swelling measurements (Figure S4) as the swelling ratio during the third cycle of pHs 6–4–6–9–6 drops significantly compared to the first cycle as can be seen in Figure 1.

The asymmetric PEM system displays a clear combination of the swelling ratio for PAH/PSS and PAH/PAA films with a slight increase in the swelling ratio at pH 9. We mainly attribute the minor increase in swelling compared to the pure PAH/PAA system to the dominant amount of PAH/PSS layers (8 bilayers). While it was shown that these kind of asymmetric films indeed form rather distinct layers,^59^ slight intermixing of the systems could influence the overall swelling degree.

#### Surface Charge

**Figure 2:**
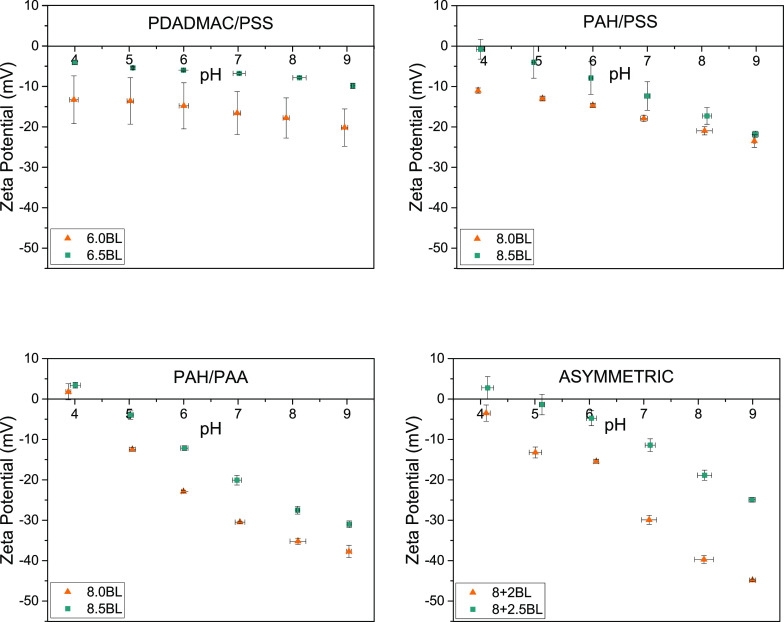
Zeta potential (mV) of negative and positive ending PEM membranes as a function of pH (−). Error bars display the standard error (sample size *n* = 3).

To obtain more information about the charge at the surface of the prepared membranes, streaming potential measurements were performed. Although retention behavior is not only determined by membrane surface charge, especially considering potential nonhomogeneous charge distributions inside PEMs,^60^ zeta potential does allow for a first indication of potential retention behavior.^61^ By determining the zeta potential at various pH values (ranging from 4 to 9), a clear influence of pH on the surface charge of the prepared membranes was observed, as shown in Figure 2.

Due to large variations in measurement values for the negative ending PDADMAC/PSS membrane, no statistically sound conclusion on zeta potential variation with pH can be made. By careful examination, one can however detect a minor decrease in the zeta potential of the positive ending PDADMAC/PSS films toward pH 9. This decrease is unexpected as the PDADMAC/PSS film should be fully ionized across the investigated pH range. In a recent work by Wang et al.,^62^ a similar drop in zeta potential with increasing pH was observed for PDADMAC/PSS-based membranes. One potential explanation could be a slight variation in surface charge caused by the adsorption of hydroxide ions with increasing pH value.^63^ As expected, the zeta potential of the membranes ending on PSS is more negative over the entire pH range.

For PAH/PSS, two clearly distinct behaviors are observed for films ending on PAH (8.5 BL, positive) and PSS (8 BL, negative). At a pH of 4, the zeta potential of positive ending films is less negative. However, at a pH of 9, the zeta potential of both negative and positive ending films match. This results in a steeper slope of zeta potential versus pH for the positive ending PAH/PSS films compared to the negative ending ones, as well as the PDADMAC/PSS films. To understand this behavior, it is important to keep the typical structure of a PEM in mind. While the charged groups of PE inside the bulk of the multilayer are compensated by mostly oppositely charged PEs (intrinsic) or ions (extrinsic), the region close to the film–solution interface consists of an excess of the final PE where the charge is not fully compensated.^64^

For the PAH ending film, the surface layer is deprotonated with increasing pH, according to its p*K*_a_ value in solution (around 8–9). The PAH inside the film that forms ionic bonds with PSS is less affected by the increase in pH, most likely due to a higher effective p*K*_a_ value, as discussed previously. While the variation of zeta potential with pH for the PAH ending film is now a combination of change in the bulk region and top layer, the film ending on PSS is only affected by the variation in bulk, since PSS is strong and therefore fully charged over the whole pH range.

Out of the symmetric PEM membranes, the PAH/PAA films display the strongest dependency of zeta potential as a function of pH, with both positive and negative ending films behaving similarly. Since for this system both PEs are weak, the difference in the top layer that was observed for the PAH/PSS system (weak/strong) is no longer present. We attribute the stronger variation in zeta potential to both PEs, varying their charge density as a function of pH. The higher absolute zeta potential at pH 9 compared to pH 4 again indicates a shift of p*K*_a_ for PAA to lower values, which could explain that no swelling was observed under these conditions.

The zeta potential of the asymmetric PEMs feature quite distinct behavior depending on the ending PE. The asymmetric layer is built from a bottom layer of 8 bilayers PAH/PSS and a top layer of 2/2.5 bilayers PAH/PAA. When ending on PAA (2 BL PAH/PAA, negative), the zeta potential variation with pH matches quite well with the one observed for the symmetric PAH/PAA system. This matches again the assumption that the zeta potential is predominantly affected by the last layer of PE, in this case, PAA. At a pH of around 5 to 6, a short plateau is observed, which could be caused by a slight shift in effective p*K* values of the PEs due to the influence of the bottom layer of PAH/PSS. When ending on PAH (2.5 BL PAH/PAA, positive), the zeta potential variation also matches that of the symmetric PAH/PAA system, although with the inflection point of the function (again related to the effective p*K* value) shifted to a high pH value.

Even though out of the studied symmetric systems, the zeta potential of the asymmetric PEMs matches the PAH/PAA system best, shifts in zeta potential as a function of pH indicate an influence of the bottom layer of PAH/PSS.

Both swelling and streaming potential measurements reveal the fundamental differences between weak and strong PEs and their incorporation in PEMs. Due to the dependency of charge density on the pH of weak PEs, the excess and surface charge of multilayers containing these is susceptible to variations in solution pH. When reaching a critical charge density, repulsive electrostatic interactions can lead to strongly enhanced swelling. For the asymmetric system, these characterization methods suggest rather distinct layers of PAH/PAA and PAH/PSS, which has been indicated in the literature.^59^ While zeta potential is dominated by the top layer of PAH/PAA (2 bilayers), the overall swelling behavior of the film is dominated by PAH/PSS (8 bilayers), due to the higher relative layer number.

### Direct Membrane Characterization

In the following section, the results of membrane performance characterization will be discussed. It should be kept in mind that the order in which these results are presented does not match the order the measurements were conducted in. Measurements where conducted in the pH order, 6–4–6–9–6. For each pH, the complete set of characterization measurements was conducted. This way, it was possible to monitor the reversibility of membrane performance, as will be discussed in the last part of this section.

#### Pure Water Permeability

The pure water permeability of a membrane is a measure of the amount of water passing through the membrane per bar of applied pressure, thus a direct indicator of the energy requirement of the filtration process. The ambition to maximize the permeability of a membrane and minimize energy consumption, however, is often limited by a decrease in solute selectivity. A somewhat simplified approach to picture this relation is by distinguishing thickness and available effective pore area as the determining factors for the pure water permeability. With a low thickness and large available effective pore area, one can achieve a high permeability. If the high available effective pore area is achieved by large pores, the membrane selectivity toward solutes is reduced.

In the case of PEM membranes, the water uptake (swelling) of the film is expected to influence both the thickness and the available effective pore area.

The ellipsometry measurements conducted in this study display an increase in swelling for PEM membranes that contain PAH/PAA (weak/weak). Therefore, one might expect an influence of solution pH on the pure water permeability of those membranes.

**Figure 3:**
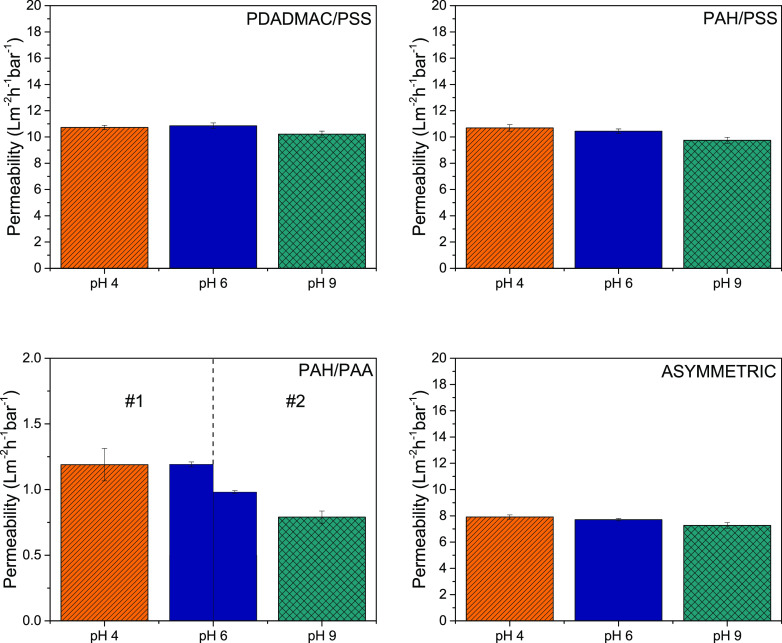
Pure water permeability (L m^2–^ h^–1^ bar^–1^) of negative ending PEM membranes as a function of feed solution pH (−). Mind the scale for PAH/PAA. Error bars display the standard error (sample size *n* = 4).

In our study, we determine pure water permeability directly at the natural pH of Milli-Q water (around 5.8) and indirectly at pH 4 and 9 from salt retention measurements by accounting for osmotic pressure. For reasons of clarity, in Figure 3 only the obtained values for the negative ending films of the studied PEM systems are displayed. Although the absolute values of pure water permeability of the positively ending films is slightly different from the negatively ending ones, the variation as a function of pH behaves in the same manner and can be found in Figure S5.

First, it should be mentioned here that due to limited long-term stability of membranes, the displayed permeability of PAH/PAA at a solution pH of 9 was measured for a different batch of membranes than at pH 4 and pH 6. The new batch has a slightly lower permeability at pH 6, around 1 L m^2–^ h^–1^ bar^–1^. The difficulties in reproducing the exact membrane properties originate from the high sensitivity of PAH/PAA multilayer growth on solution pH in the range from pH 5 to 6.^24,26^ In the range of pH 4 to 9, we do not observe a sharp increase in pure water permeability as a function of pH. This indicates that all PEM systems do have a stable film structure in that range. Overall, there is a slight trend of decreasing permeability with increasing pH. This could be caused by long-term aging effects of the PEM layers leading to densification of the film (after all, these films are not in thermodynamic equilibrium). However, an alternative explanation could be slight fouling over the course of the filtration measurements. The much bigger effect on the PAH/PAA membranes might be caused by increased swelling of the film at high pH values, which was also observed at pH 9 for PAH/PAA films as shown in Figure 1.

There are clear differences in pure water permeability at pH 6 between the different systems. The lowest permeability is observed for the system PAH/PAA (1–1.2 L m^2–^ h^–1^ bar^–1^), about one magnitude lower compared to the other systems, which is mostly related to the much higher film thickness (see Table 1). To a certain extent, the mesh size of the PEM system might play a role in determining the pure water permeability, which was shown by Krasemann et al.^65^ to be related to charge density of PE used. In this case, PAH/PAA has the highest charge density of the studied PEM systems and is therefore expected to have the smallest mesh size. We will discuss this in more detail when evaluating the MWCO.

The permeability of PAH/PSS (10 L m^2–^ h^–1^ bar^–1^) is slightly lower than the one of PDADMAC/PSS (11 L m^2–^ h^–1^ bar^–1^). Even though PDADMAC/PSS forms thicker layers on a model surface (see Table 1), which should result in a lower pure water permeability, it should be kept in mind that the amount of bilayers necessary to obtain a defect free layer is less (only 6 bilayers compared to 8 bilayers for PAH/PSS).

In addition, the charge density of PAH/PSS (0.091 ion pairs in the complex/carbon atoms in the complex) is higher compared to PDADMAC/PSS (0.063 ion pairs in the complex/carbon atoms in the complex), therefore the system is likely to form more dense layers.^65^

The asymmetric PEM system based on PAH/PSS and PAH/PAA (permeability of around 8 L m^2–^ h^–1^ bar^–1^) has a reduced permeability compared to PAH/PSS, caused by the extra 2 bilayers of PAH/PAA coated on top.

#### Molecular Weight Cutoff

The second measure of membrane performance is the MWCO, which describes the selectivity of the membrane toward solutes based on size exclusion (here determined from PEG retention). Similar to pure water permeability, one might expect an influence of solution pH on size-based exclusion due to swelling. An increasing amount of water inside the film might, in addition to increasing the thickness of the film, lead to an increase of the mesh size and with that a reduced steric hindrance for solutes.

**Figure 4:**
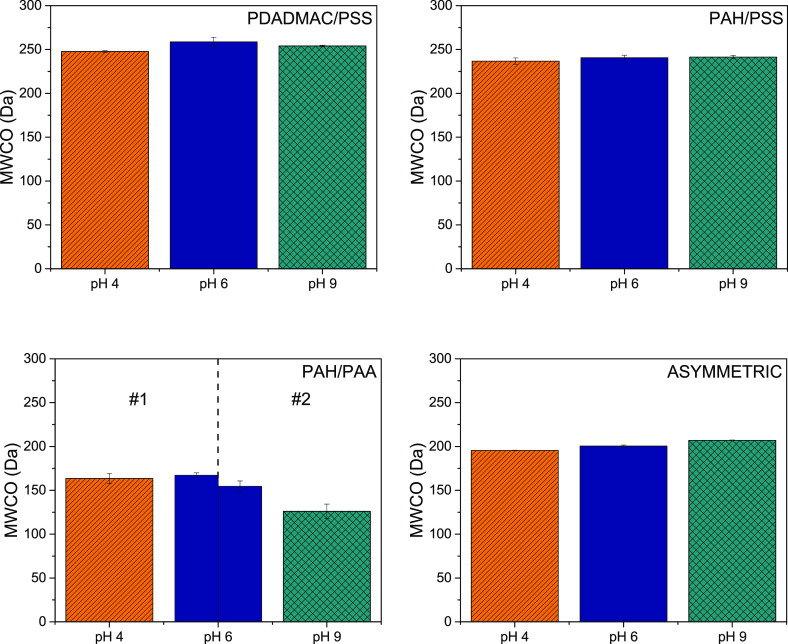
MWCO (Da) of negative ending PEM membranes as a function of feed solution pH (−). Error bars display the standard error (sample size *n* = 4).

The determined MWCO of the negative ending films at solution pH values of 4, 6, and 9 are shown in Figure 4. Similar to pure water permeability measurements, the results of MWCO for positive ending films are qualitatively similar to the negative ending once and can be found in Figure S6. Furthermore, sieving curves of the initial measurement at pH 6, after exposure to pH 4 and after exposure to pH 9 can be found in Figure S7.

For the PAH/PAA system, the graph again distinguishes between the first and second batch of membranes, as discussed previously. There is no significant change in MWCO for the three PEM systems PDADMAC/PSS, PAH/PSS, and the asymmetric multilayer as a function of solution pH. This is in accordance with the minor changes in swelling and pure water permeability observed for these systems.

Similar to the pure water permeability, the MWCO of PAH/PAA decreases at pH 9. This indicates a “densification” of the membrane, meaning a reduction in effective pore size. This is counterintuitive to the idea of swelling of the multilayer, as observed for pH 9 in Figure 1, leading to thicker but more open films. Considering, however, the observed permanent reduction in wet thickness of the PEM after exposure to pH 9 (Figure S4), we suspect the polyelectrolyte chains to restructure in an energetically more favorable and more dense configuration. One can additionally imagine that the induced mobility at pH 9 for the membrane promotes “filling” of defects within the layer (which has been observed even for high bilayer numbers of PAH/PAA^15^) due to the expansion and reorganization of the PE chains at high pH. However, further research is needed to confirm this theory.

Although the MWCO is a process parameter of a membrane (meaning it is dependent on parameters such as the flux through the membrane), it gives a good estimate of the mesh size of the membrane, especially for similar fluxes. Comparing all films at pH 6, the films are in the order, [PDADMAC/PSS] > [PAH/PSS] > [asymmetric] > [PAH/PAA], where a high MWCO represents a large mesh size. For the symmetric systems, this matches nicely considering the charge density of PE complexes:^65^ [PDADMAC/PSS] < [PAH/PSS] < [PAH/PAA]. For the asymmetric system, one would expect a MWCO that is similar to the symmetric PAH/PAA system. The fact that the MWCO is between the symmetric systems of PAH/PSS and PAH/PAA suggests that the PAH/PAA layer is not fully developed, yet. Since the MWCO would typically slightly decrease with an increasing flux, the MWCO of the PAH/PAA film at comparable flux would be even lower. However, this would not change the observed order in MWCO between the systems.

#### Salt Retention

The retention of salts by NF is mostly determined by electrostatic interactions between the ions and the membrane. Here, commonly two effects are distinguished: Donnan exclusion describes the interaction between charged groups of the membrane and the ions, and dielectric exclusion describes the interaction between induced charges at the interface of two media with different dielectric properties (here the aqueous and membrane phase). Those two effects can be distinguished by their effect on salt retention. Donnan exclusion causes salts that contain co-ions (same charge as the membrane) with higher valence to be repelled to a larger extent, which because of electroneutrality also leads to high retention of the counterion. Dielectric exclusion causes any salt with ions of high valency, independent of sign, to be retained to a larger extent. Therefore, salt retention measurements of binary mixtures with different combinations of mono- and divalent ions can give qualitative information on membrane charge and exclusion mechanisms (assuming that the influence of salts on membrane properties are minor).

**Figure 5:**
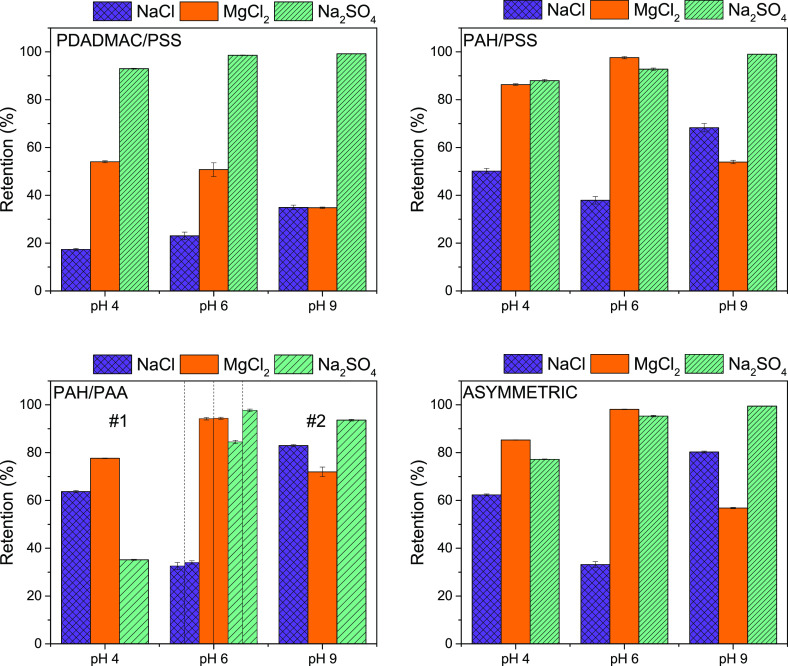
Salt retention (%) determined via conductivity for negative ending PEM membranes as a function of feed solution pH (−). Four PEM systems: [PDADMAC/PSS]6 (top left), [PAH/PSS]8 (top right), [PAH/PAA]8 (bottom left), [PAH/PSS]8+[PAH/PAA]2 (asymmetric, bottom right). Bars display the retention of NaCl (left), MgCl_2_ (middle), and Na_2_SO_4_ (right) for each pH and system. Error bars display the standard error (sample size *n* = 4).

In Figure 5, we show retention of three salts (NaCl, MgCl_2_, and Na_2_SO_4_) determined for the negative ending PEM systems at different pH. Retention values are based on conductivity measurements of permeate and feed samples, where the influence of HCl and NaOH on the conductivity is neglected due to the at least 50-fold higher concentration of salt compared to added acid/base. Again for reasons of clarity, the retention values for positively ending PEM systems are presented in Figure S8. It should be mentioned, that there is a clear difference in salt retention for the PEM system with a different top layer at the same pH value. Here, salt retentions indicate more positive effective charge values for positive ending layers. However, the trend with variation as a function of pH is again similar.

The PDADMAC/PSS system, surprisingly, displays a clear trend in decreasing MgCl_2_ retention, increasing NaCl as well as increasing Na_2_SO_4_ retention with pH. This indicates a slight variation in effective membrane charge with pH, although no charge reversal is observed. This matches qualitatively to the observed decrease in zeta potential with increasing pH (Figure 2). Across all pH values the PDADMAC/PSS film displays an overall negative membrane charge with high retention toward Na_2_SO_4_ combined with lower retentions for NaCl and MgCl_2_, indicating that salt retention is dominated by Donnan exclusion.

For the PAH/PSS films, typical dielectric exclusion-dominated retention behavior is observed at pHs 4 and 6 since both MgCl_2_ and Na_2_SO_4_ retention are high. Although MgCl_2_ retention unexpectedly decreases at pH 4, general trends in salt retention indicate the transition of a slightly positive membrane at pH 4 to a negatively charged membrane at pH 9. With a p*K*_a_ value between 8 and 9, PAH will be ionized for approximately 50% at pH 9.^25^ The reduced ionization degree of PAH at pH 9 leads to a more negative charge in the PAH/PSS film.

The reduced MgCl_2_ retention at pH 9, compared with pH 4 and 6, could therefore very well be explained by an increase in negative charge on the membrane and thus by a shift toward Donnan exclusion at pH 9. It should be highlighted, that although the respective variation in zeta potential for PDADMAC/PSS and PAH/PSS is quite similar (Figure 2), the variation in effective charge (indicated by salt retention) is more severe for PAH/PSS. This can be again explained by distinguishing the surface and bulk charge of the PEM. Since both films end on PSS, the surface potential is similar. The bulk charge, however, is for the PAH/PSS film affected by deprotonation of PAH.

Like in Figures 3 and 4, the data distinguishes between the first and second batch of PAH/PAA membranes. Being a system comprised of two weak PEs, a clear distinction is expected with regard to membrane charge at pHs 4 and 9. At pH 4 one can see the PAH/PAA film acts as a positively charged layer with high retention for MgCl_2_ combined with low retention for Na_2_SO_4_, which is typical for Donnan exclusion dominated retention. This matches with the p*K*_a_ value of PAA (6.5) and PAH (8–9), meaning at pH 4, PAA is deionized whereas PAH is fully ionized.^25^ Again, the reduced MgCl_2_ retention compared to pH 6 is surprising as one would expect a much higher value (considering the other salt retentions). The exact opposite can be seen at pH 9, where the PAH/PAA film displays a more negative charge due to the partial deionization of PAH and the retention for Na_2_SO_4_ is higher than that for MgCl_2_. It also stands out that the retention for all salts at pH 9 is very high. This matches to the sudden densification of the membrane that was observed at pH 9 (see Figures 3 and 4). With a minimum in NaCl retention at pH 6, and at the same time very high retention toward both divalent salts, the salt retention seems to be dominated by Dielectric exclusion, indicating a minimum in effective charge density. This is unexpected considering the p*K*_a_ values of the PEs in solution, but one has to keep in mind that a shift of the p*K*_a_ value is observed in the literature when PEs are incorporated in multilayers.^58^ This explains the close to neutral effective charge, since both PEs would be fully charged (complete charge compensation).

Lastly, it can be seen in Figure 5 that the retention behavior of the asymmetric system matches with the PAH/PSS film, especially at pHs 6 and 9. At pH 4, the Na_2_SO_4_ retention is lower (approximately 10%) and NaCl retention is higher compared with the PAH/PSS film; this is believed to be caused by the presence of the 2BL PAH/PAA film. From this it can be concluded that the more open bottom layer (PAH/PSS) still plays a big role with regard to salt retention of the asymmetric system. This clearly displays the difference in the two measurement methods (streaming potential and salt retention), again, and strongly suggests a distinct layer structure of these multilayers. While the top layer of PAH/PAA clearly dominates the zeta potential (Figure 2), the overall salt retention is dominated by the thicker PAH/PSS support layer. This is something that has to be kept in mind when using zeta potential measurements to predict salt retention, especially for this type of PEM. Depending on the layer structure, zeta potential values might be misleading as an estimate of effective charge of the selective membrane layer.

One system that displayed unexpected and partially inexplicable results is PDADMAC/PSS, both the positive and negative ending membrane (see Figure S6). Both display significant changes in salt retention with varying pH and low reversibility of retention behavior at pH 6 after exposure to pHs 4 and 9. Considering streaming potential results and the fact that both PDADMAC and PSS are considered strong PEs (so fully charged over the whole pH range) only minor fluctuations in salt retention were expected.

To check for potential retention of hydrogen and hydroxide ions and the associated influence on the retention measured via conductivity, the pH of permeate samples was monitored for filtration measurements at pH 4 and pH 6. As measurements were conducted in open atmosphere, at pH 9, the solution pH would drop slowly over time due to the incorporation of carbon dioxide from the atmosphere to form bicarbonate. The solution was therefore manually titrated with NaOH to keep the pH stable. The addition of a buffer was avoided since it might influence the retention behavior. Caused by the length of the filtration measurement, the pH of permeate samples would already drop quite severely, making it impossible to estimate the transport of hydroxide ions through the membrane in this case. The difference of pH between permeate and feed phase is shown in Figure S9. A clear trend in pH variance, and with that in the transport behavior of hydrogen ions, is observed for different salts depending on the retention of the dominant salt. This matches nicely to the theory of negative retentions and the influence of a dominant salt in NF discussed in detail by Yaroshchuk.^66,67^ In short, the electric field created by the retention of especially Na_2_SO_4_ and MgCl_2_ causes the much more mobile hydrogen atoms to be either slowed down (higher pH permeate side) or accelerated (lower pH permeate side), respectively. Knowing the diffusion coefficient, charge, and concentration of the ions that are present, the influence on electrical conductivity can be estimated using the following formula:^68^
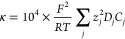
8with κ being the conductivity in μS cm^–1^, *F* the Faraday constant, *R* the ideal gas constant, *T* the temperature (297.15 K), *z*_*j*_ the charge of the ion, *D*_*j*_ the diffusion coefficient of the ion (m^2^ s^–1^), and *C*_*j*_ the concentration of the ion (mol m^–3^). Using this equation, one can evaluate the influence of hydrogen or hydroxide ions on the overall conductivity used to estimate the retention.

Here we show the potential influence for three extreme cases, one of which partially explains the unexpected behavior of decreasing MgCl_2_ retention at pH 4 compared to pH 6. The first case is a positive membrane with a high retention of 90% for MgCl_2_ at pH 4. As a consequence, the pH in the permeate will decrease (also measured, estimated around 3.5). Neglecting the influence of pH on conductivity, this would correspond to a conductivity of around 1320 μS cm^–1^ in the feed and 130 μS cm^–1^ in the permeate. Considering the influence of pH, the feed conductivity is around 1360 μS cm^–1^, and the permeate conductivity is around 270 μS cm^–1^. This results in an observed retention of only 80%. This matches well with the observed drop in retention of MgCl_2_ when going to pH 4 for almost all PEM systems. Considering the uncertainty in permeate pH measurements during MgCl_2_ retention, one can estimate a range for the retention variation from −30% to −10% for a permeate pH of 3 and 3.5, respectively. The second case considers the retention of NaCl at pH 4. Here we assume a retention of 30%. As also shown in the measurements, no significant variation in pH of the permeate is expected. With the same calculation as before, the observed retention only shows a minor drop to around 28%. The third case considers a negative ending membrane with a high retention of 90% for Na_2_SO_4_ at pH 9. On the basis of the observed pH variations, the permeate pH is expected to be around pH 10. With the use of, again, eq 8, this results in an observed retention of 88%. Therefore, it is shown that the presence of hydrogen/hydroxide ions only influences the observed retention based on conductivity measurements for the extreme cases of high magnesium chloride retention at pH 4. Variation of salt retention for the PDADMAC/PSS membrane can not be explained by the influence of pH on conductivity measurements. As for a strong PEM system, no variation in charge density with pH is expected, and the significant variations observed are unexpected and leave us puzzled.

The evaluation of performance of NF membranes based on the studied PEMs allows the characterization of PEMs from a different point of view. Complementing these studies with the presented ellipsometry and streaming potential measurements, a more complete insight into the PEM structure is gained, as well as, strength and weaknesses of different methods are revealed. Ellipsometry measurements clearly indicate changes in the cross-linked network structure of the PEM caused by variation in charge density of PEs. However, direct translation to membrane properties in terms of steric retention and hydraulic resistance can be difficult. Not only is the support structure of the film different (silicon wafer vs UF membrane), but also can the effect of swelling and increased chain mobility be counterintuitive.

This is illustrated in the fact that the PAH/PAA film swells a lot at a pH of 9 and ambient pressure, while the membrane permeability and MWCO both decrease, illustrating a densified structure of the selective layer (supported by salt retention measurements).

Streaming potential measurements display the variation in charge density of weak PEs with pH but are limited to indicating the surface charge of the PEM. It should be mentioned that the typical odd–even effect (typically revealed by an alternating positive and negative zeta potential) was not observed in the performed study. As we are not sure about the cause for the very little shift of absolute zeta potential when ending on a positive PE, we focus only on the quantitative variation of zeta potential with pH. Although, the variation of zeta potential matches qualitatively to the ending PE and its p*K* value, salt retention measurements reveal the quantitative offset of the method. In addition, the zeta potential is clearly misleading for the asymmetric PEM, as the salt retention is dominated by the support layer of PAH/PSS while the zeta potential is dominated by the top layer of PAH/PAA.

#### Reversibility

To monitor for irreversible changes to the membrane performance after exposure to pHs 4 and 9, measurements at these pH values are followed up by measurements at pH 6 (whole sequence pHs 6–4–6–9–6). Since PEMs are not necessarily in their energetically most favorable conformation (kinetically limited state), one might expect reorganization of chains given sufficient mobility, for example at a pH that reduces ionic interaction by partial deionization.

As well, PEMs based on weak PEs might fall apart in certain pH conditions, but this is not expected for the studied pH range.

**Figure 6:**
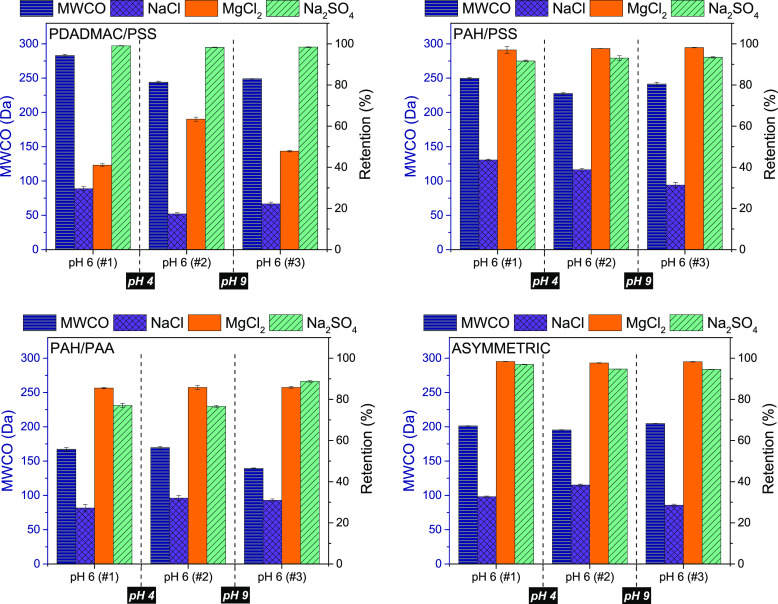
Reversibility behavior of negative ending PEM membranes during MWCO (Da) (left *y*-axis) and salt retention (%) (right *y*-axis) experiments as a function of pH (−). Error bars display the standard error (sample size *n* = 4).

Salt retention (NaCl, MgCl_2_, and Na_2_SO_4_) as well as MWCO at pH 6 for negative ending PEMs is displayed in Figure 6. For reasons of clarity, measurements of permeability, as well as the full set for positive ending layers, are shown in Figures S10, S11, and S12.

Since the filtration performance of most systems is quite stable over the series of measurements, we we will only discuss significant deviations in the following. PDADMAC/PSS is a combination of two strong PEs and is therefore expected to not show any irreversible changes over the tested pH range.^18^ Surprisingly, PDADMAC/PSS displays significant variations in MWCO, NaCl, and MgCl_2_ retention after filtration at pH 4. After filtration at pH 9, the variation at pH 4 is counteracted to a certain degree, and initial conditions however are not restored. Considering the good reversibility of the other systems, we are unsure about the reason for these deviations. One explanation for the variation in salt retention could be the dominance of Donnan exclusion for this system at pH 6, which is very much determined by membrane charge and thus more sensitive to slight variations in charge density. The drop in MWCO after the first measurement at pH 4 is currently without a clear explanation. The salt retention of PAH/PSS on the other hand is very stable with only a slight continuous decrease of NaCl retention.

Also, PAH/PAA displays good reversibility after pH 4. For reversibility after pH 9, we have to take into account the fact that a new batch (#2) of PAH/PAA membranes was used. These had a slightly higher Na_2_SO_4_ retention at pH 6 and a slightly lower MWCO compared to batch #1. With regard to salt, we observe nice reversibility in contrast to MWCO. The MWCO drops to approximately 125  Da at pH 9 after which it increases again toward 140 Da but seems not to reach the original value of 167 Da. This could possibly be due to rearrangement of polymer chains at pH 9 as indicated by ellipsometry measurements (see Figure S4), resulting in densification of the PAH/PAA film and/or closing of possible defects which are known to be present in PAH/PAA films.^15^

Finally, the asymmetric system shows reversible behavior after both pH 4 and 9. It has to be mentioned here that the positive ending membranes of the displayed systems, especially considering salt retention, display worse reproducibility than the negative ending ones. However, they also display a clear trend of decreasing effective membrane charge density over the run of the measurement series (increasing Na_2_SO_4_ retention, decreasing NaCl and MgCl_2_ retention). We attribute this to fouling, which is known to be more severe for positive membranes.^69−71^ Here, negatively charged foulants are expected to deposit at the membrane surface and therefore reduce the effective charge density.

## Conclusions

PEM NF membranes were demonstrated to be susceptible to solution pH, depending on the nature of PEs. Four fundamentally different systems were systematically studied for changes in steric network structure as well as excess charge of the multilayer. PEMs purely based on strong PEs (PDADMAC/PSS) are surprisingly enough to a small degree susceptible to solution pH. As both PEs are fully charged over the studied pH range, one would not expect an influence of pH on PEM properties. Relatively, minor decrease in charge density with increasing pH (that have been previously observed in literature) are attributed to hydroxide ion adsorption and affect salt retention behavior. Still these membranes stay negatively charged over the whole pH range.

Exchanging one strong PE (PDADMAC) for a weak PE (PAH), results in a severe dependency of PEM charge as a function of solution pH. This is due to the partial deprotonation of PAH at increasing pH values, which leads to an excess negative charge. One consequence is the variation of single salt retention of membranes with solution pH. The network structure of the PEM, however, is unaffected by pH in the studied range. Going to a system that is based on two weak PEs (PAH/PAA), an even stronger variation of PEM properties is observed. Since, the charge densities of both PEs are affected by solution pH, the variation of excess charge of the PEM is larger. In this case, an additional reorganization of the PEM network structure is observed. Ellipsometry measurements on a model surface reveal that excessive swelling at first exposure to pH 9 is followed by permanently reduced swelling in the studied pH range (including repeated exposure to pH 9). Considering the permanent decrease in MWCO and pure water permeability of the membrane after exposure to pH 9, we conclude an effective densification of the selective PEM layer.

Lastly, an asymmetric PEM that is based on PAH/PSS as a support layer and PAH/PAA as a dense top layer was studied. Also, this PEM is susceptible to solution pH. One can distinguish effects of the top layer, which influences zeta potential, from the bottom layer, which dominates salt retention. The MWCO for this system does not vary significantly with solution pH. The fact that the MWCO lies between the one that is observed for the symmetric systems suggests that the PAH/PAA layer is not fully developed, yet.

## Supporting Information Available

Additional data with regard to PEM membranes ending on a positively charged layer, similar to the data displayed for the negatively ending PEMMs; it includes membrane module design, a schematic illustration of the experimental crossflow setup, and an example of the obtained sieving curves resulting from MWCO measurements; and lastly, it contains data on the pH difference between permeate and feed solutions over single salt retention (PDF)
