# Supplementary material for: Effects of Feed Solution pH on Polyelectrolyte Multilayer Nanofiltration Membranes
Source: ACS Appl Polym Mater. 2022 Dec 20;5(1):355–69. doi: 10.1021/acsapm.2c01542 (PMC9841528; doi:10.1021/acsapm.2c01542)
Supplement: Supplementary file 1 — ap2c01542_si_001.pdf [file ap2c01542_si_001.pdf]

Supporting Information

Effects of Feed Solution pH on Polyelectrolyte Multilayer Nanofiltration Membranes

Moritz A. Junker,[†] Jurjen A. Regenspurg,[†] Cristobal I. Valdes Rivera,[‡,†] Esra te Brinke,[†] and Wiebe M. de Vos[*,†]

†*Membrane Science and Technology, University of Twente, MESA+ Institute for Nanotechnology, P.O. Box 217, 7500 AE Enschede, the Netherlands*

‡*International Institute for Infrastructural, Hydraulic and Environmental Engineering—IHE, P.O. Box 3015, 2601 DA Delft, The Netherlands*

E-mail: w.m.devos@utwente.nl

Figure S1: Crossflow schematic

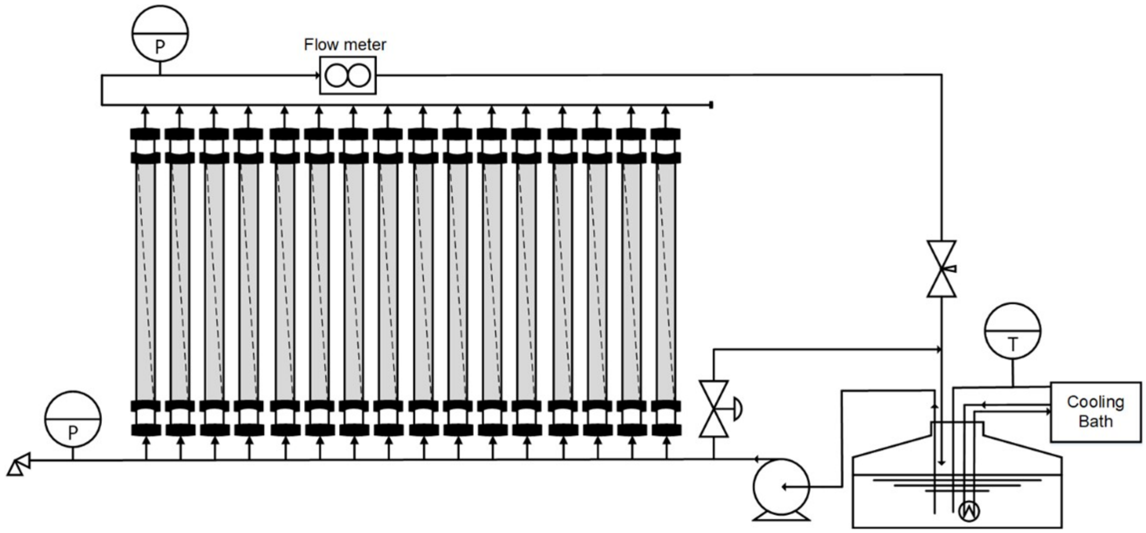

Figure S1: Schematic representation of the used crossflow setup.

Feed solution was pumped through a maximum of 16 membrane modules in the crossflow setup. Depending on the type of measurement, e.g. pure water permeability, salt retention or MWCO, feed solution was permeated through the membranes for at least 30 min to ensure stable permeation before starting the measurement. A transmembrane pressure of 5 bar over the membranes was created using a needle valve outlet in combination with pump frequency. The trans membrane pressure was monitored using pressure sensors before and after the membrane modules. The flowrate was monitored using a flow meter which was placed after the membrane modules. Feed temperature was kept constant at approximately 20 °C using a cooling spiral in the feed solution. All crossflow measurements were performed for at least 1 hour to ensure representative permeate samples. Feed solution pH was continuously monitored using a FiveEasy^{TM} benchtop pH meter from Mettler-Toledo B.V. ***The schematic representation was obtained from and used with permission of the authors of Elshof et al. (2020) doi: https://doi.org/10.1016/j.memsci.2020.118532***.

Figure S2: Membrane module

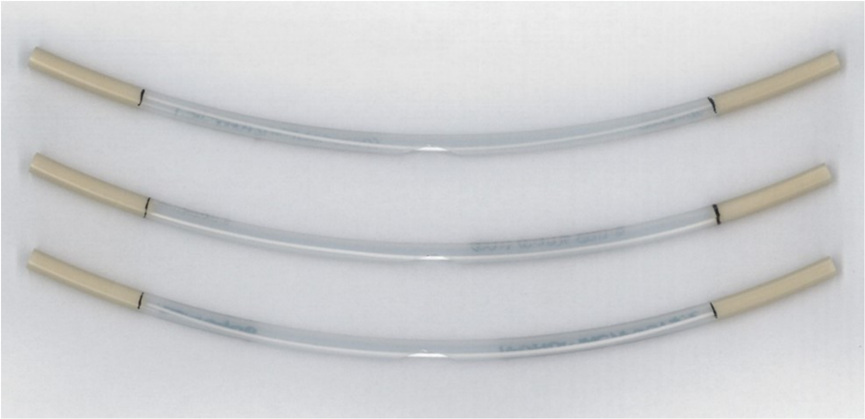

Figure S2: Membrane module.

The modules are fabricated from 6 mm tubing. A single hollow fiber was placed inside the 6 mm tubing after which the outer ends are potted using a 2 component polyurethane glue. At the mid point of the membrane module an opening is present in the tubing which allows for the collection of permeate. ***Picture was adapted from Elshof et al. (2020) doi: https://doi.org/10.1016/j.memsci.2020.118532***.

Figure S3: Normalized RI signal and Sieving curve

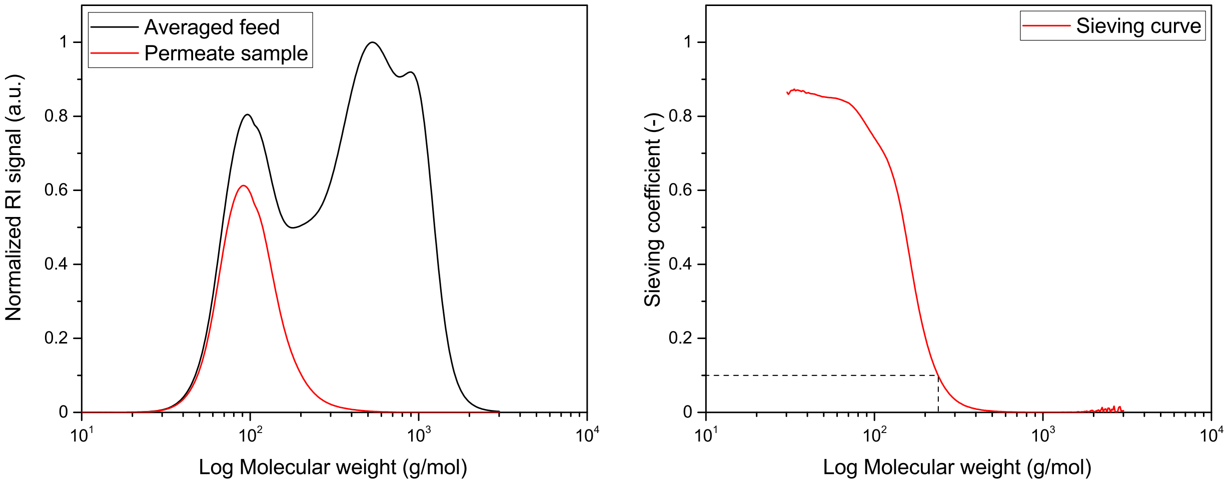

(a) (b)

Figure S3: (a) Example of normalized RI detector signal vs. Mw (g/mol) for a permeate sample and averaged feed. Feed samples were taken at the start and end of each measurement. (b) Resulting sieving curve obtained from the data displayed in figures S5 and S10. The 90 % MWCO is indicated with the dashed line.

Figure S4: Swelling ratio as a function of pH

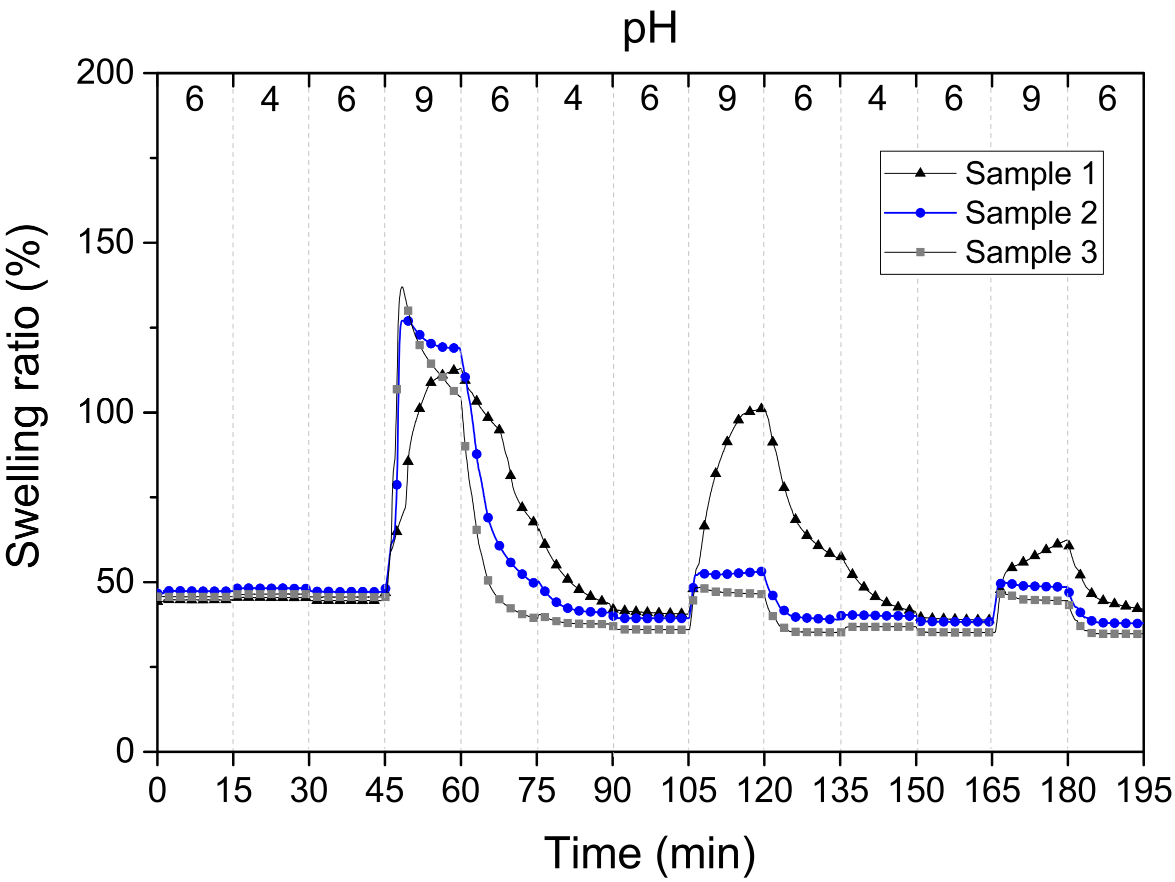

Figure S4: Multilayer swelling ratio (%) as a function of pH (-) over time (min). The swelling ratio was obtained by means of Ellipsometry as described in the methods section.

Figure S5: Pure water permeability positive ending membranes

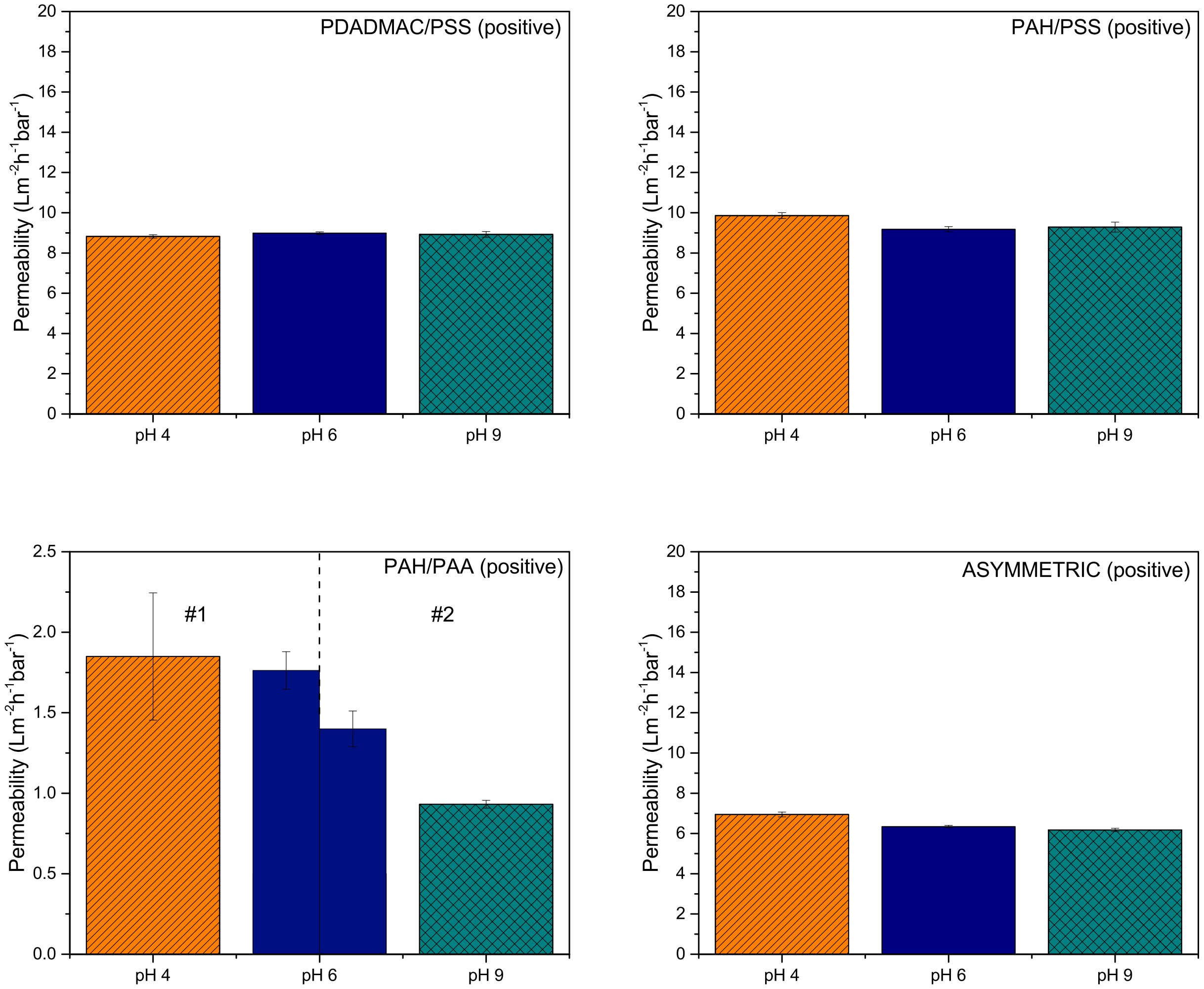

Figure S5: Pure water permeability ($Lm^{-2}h^{-1}bar^{-1}$) of positively ending PEM membranes as a function of feed solution pH (-). Mind the scale for PAH/PAA. Error bars display the standard error (sample size n=4).

Figure S6: Molecular Weight Cut-off positive ending membranes

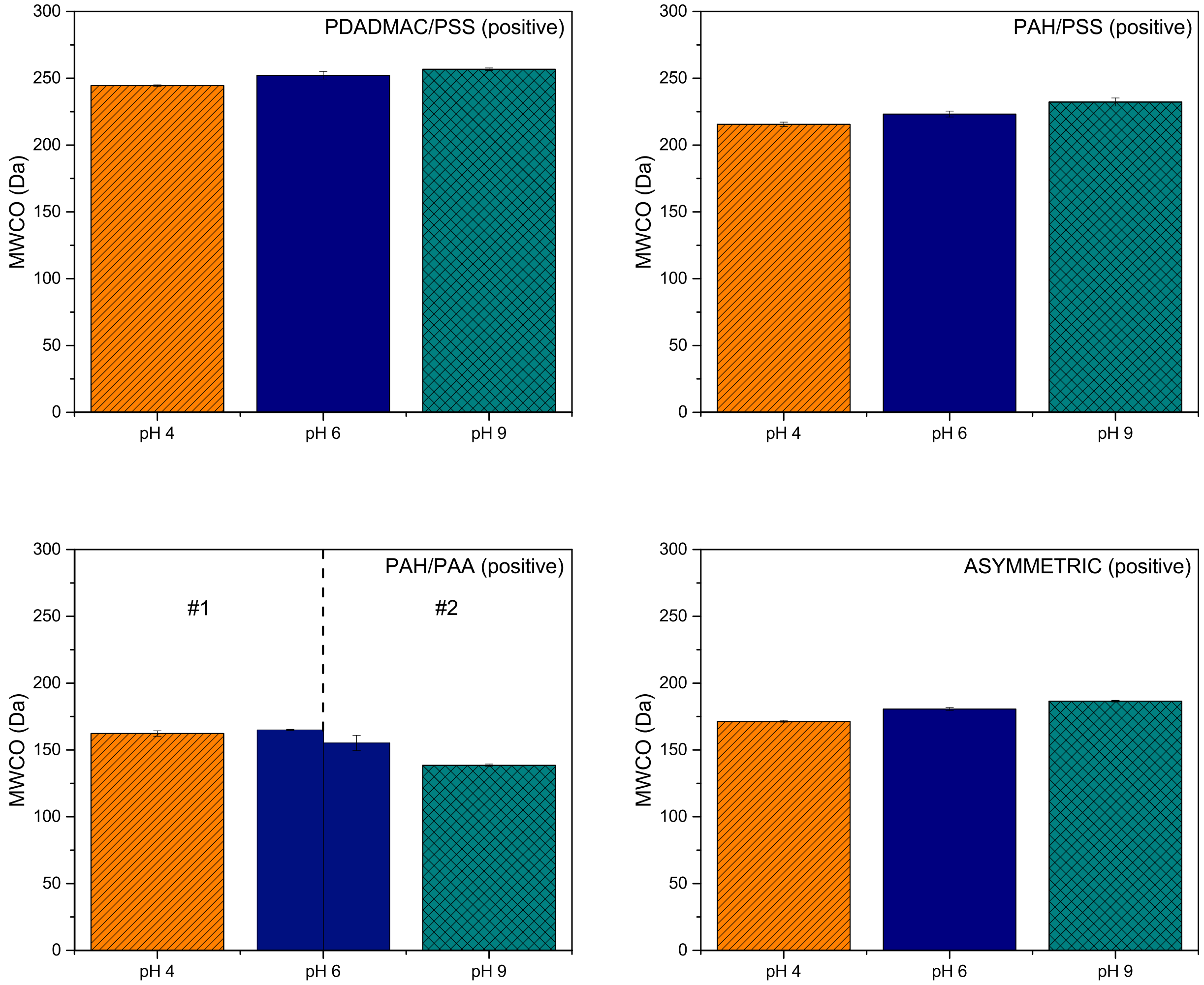

Figure S6: MWCO (Da) of positively ending PEM membranes as a function of feed solution pH (-). Error bars display the standard error (sample size n=4).

Figure S7: Molecular Weight Cut-off Sieving Curves

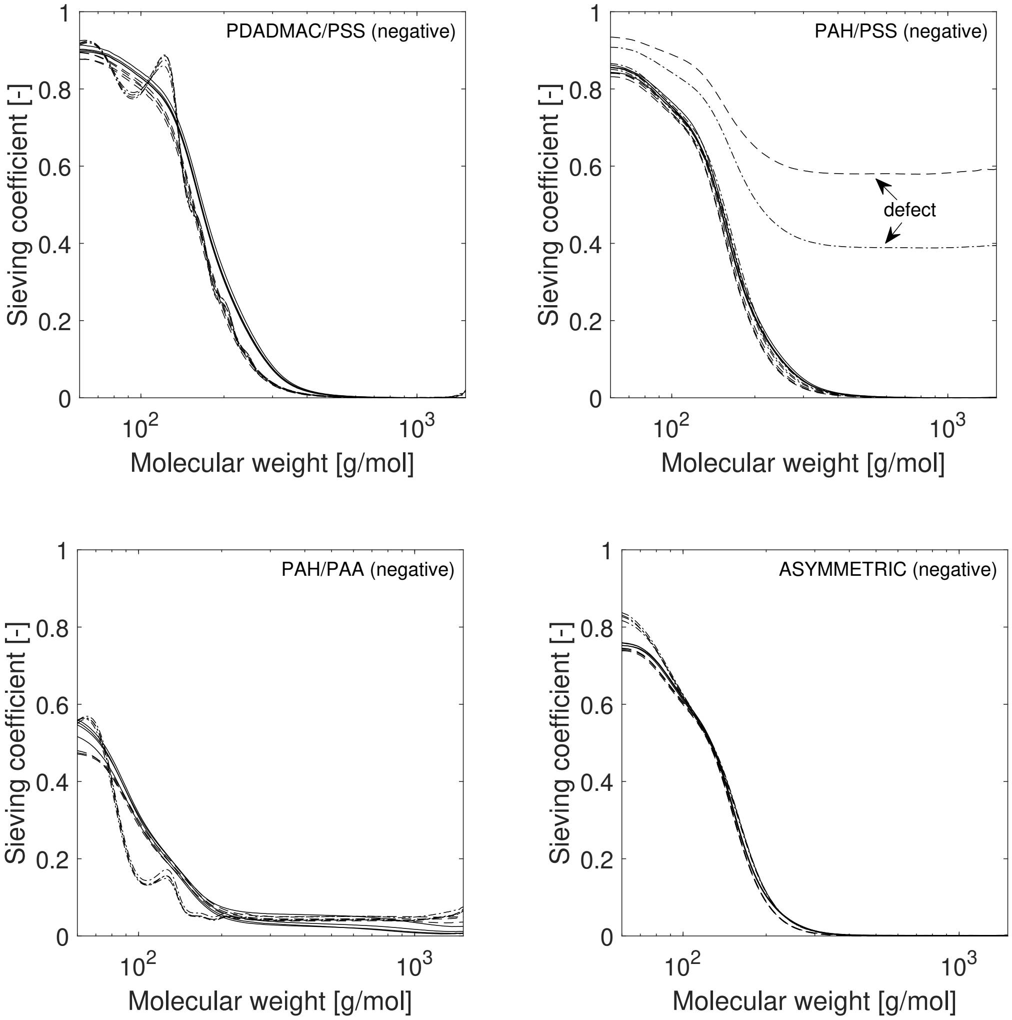

Figure S7: Sieving coefficient (-) of negatively ending PEM membranes at pH 6. Initial measurement (-), after exposure to pH 4 (–) and after exposure to pH 9 (-.).

Figure S8: Salt retention positive ending membranes

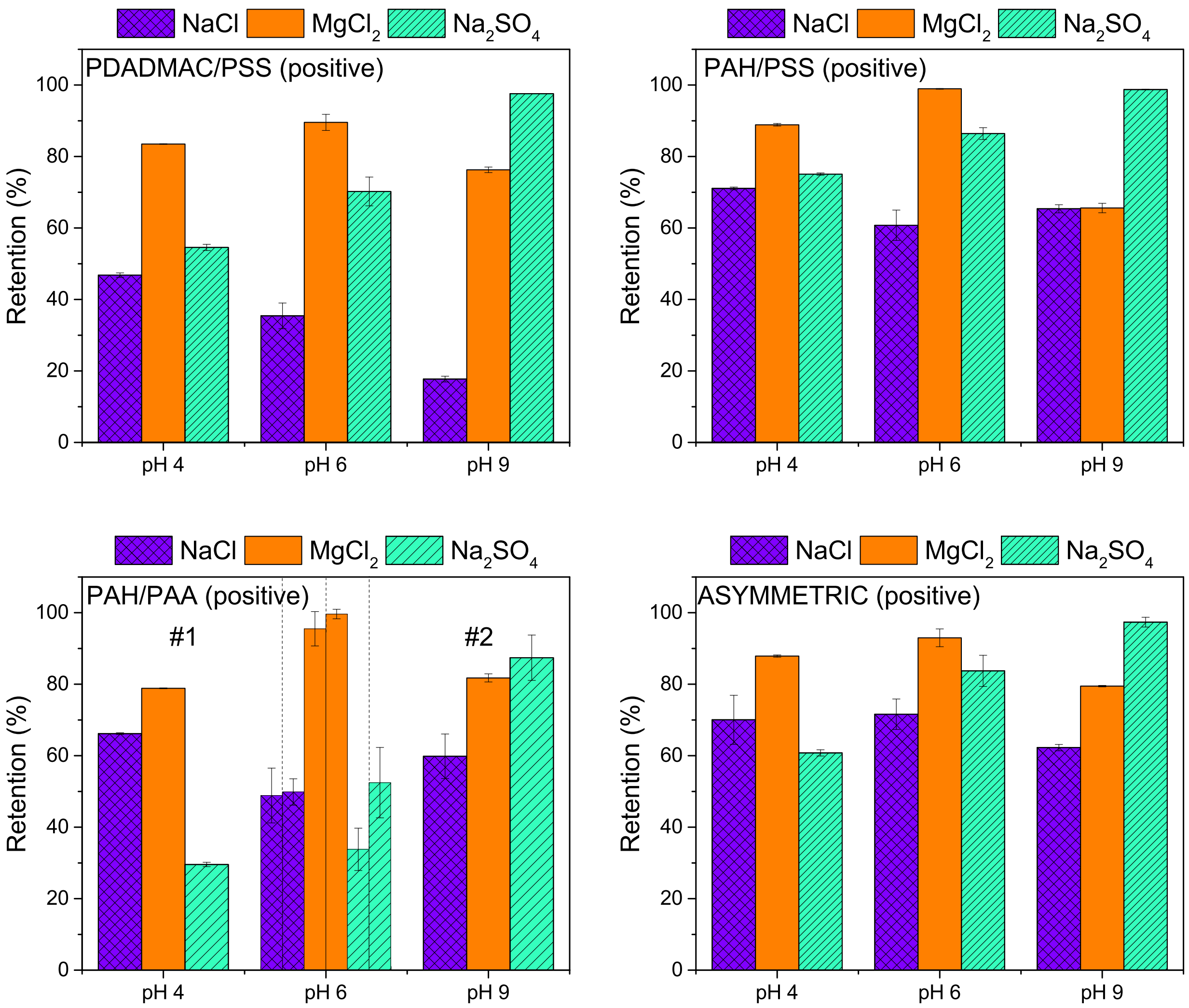

Figure S8: Salt retention (%) of positively ending PEM membranes as a function of feed solution pH (-). Error bars display the standard error (sample size n=4).

Figure S9: pH difference in single salt retention

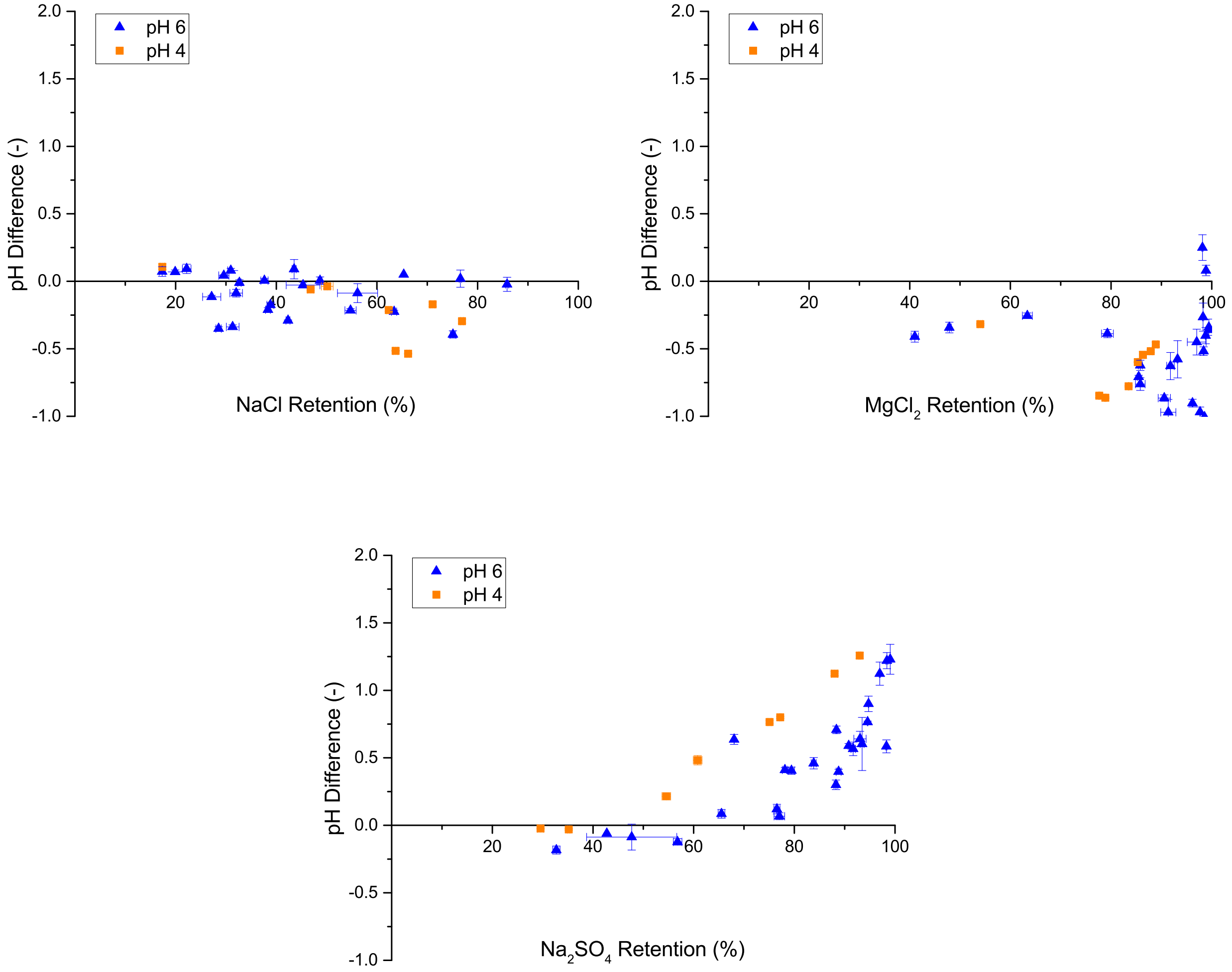

Figure S9: pH difference between permeate and feed solution over single salt retention (%) for all membranes at pH 4 and pH 6. Error bars display the standard error (sample size n=4).

Figure S10: Pure water permeability reversibility positive ending membranes

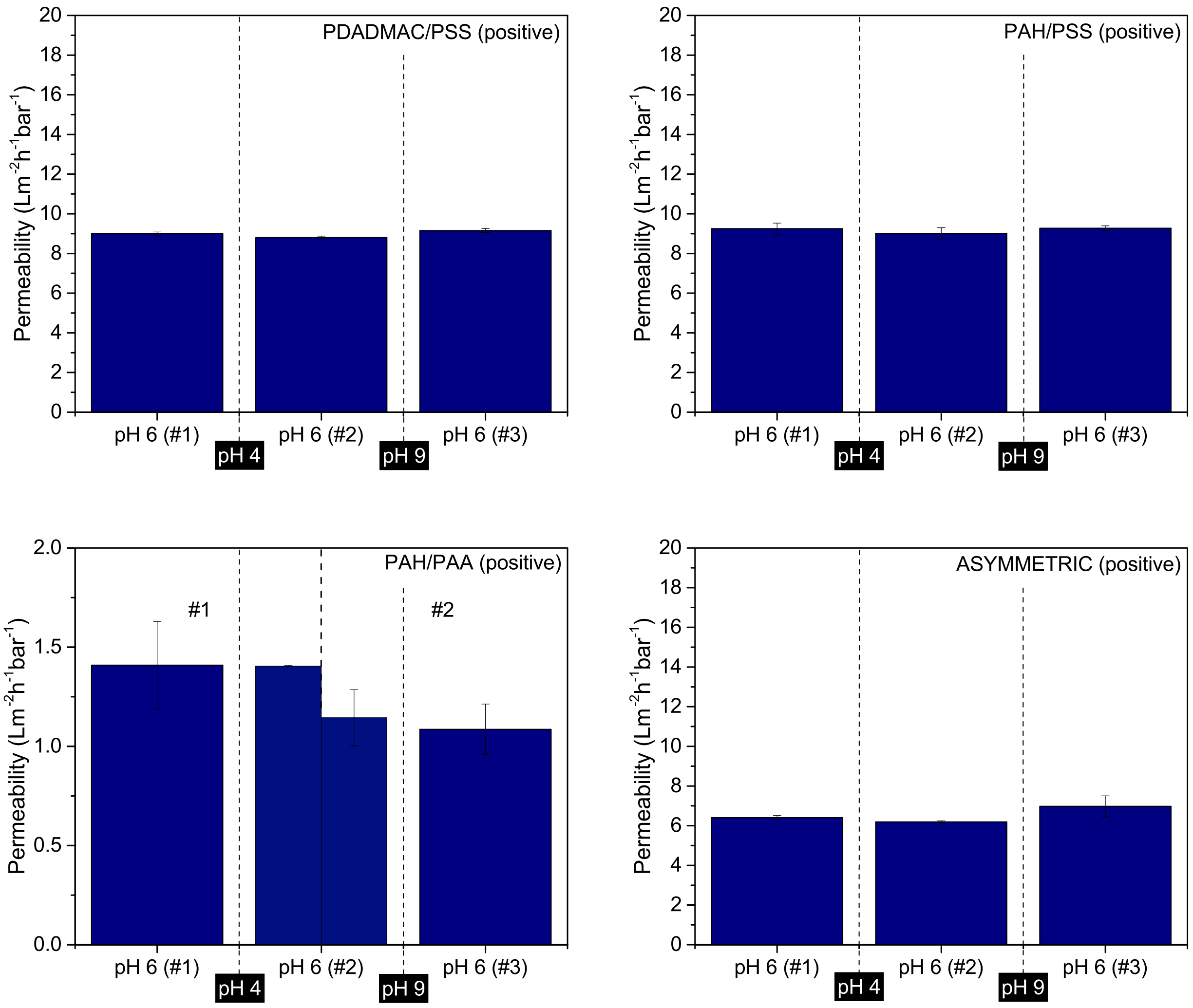

Figure S10: Reversibility behavior of positively ending PEM membranes during pure water permeability ($Lm^{-2}h^{-1}bar^{-1}$) experiments as a function of feed solution pH (-). Mind the scale for PAH/PAA. Error bars display the standard error (sample size n=4).

Figure S11: Pure water permeability reversibility negative ending membranes

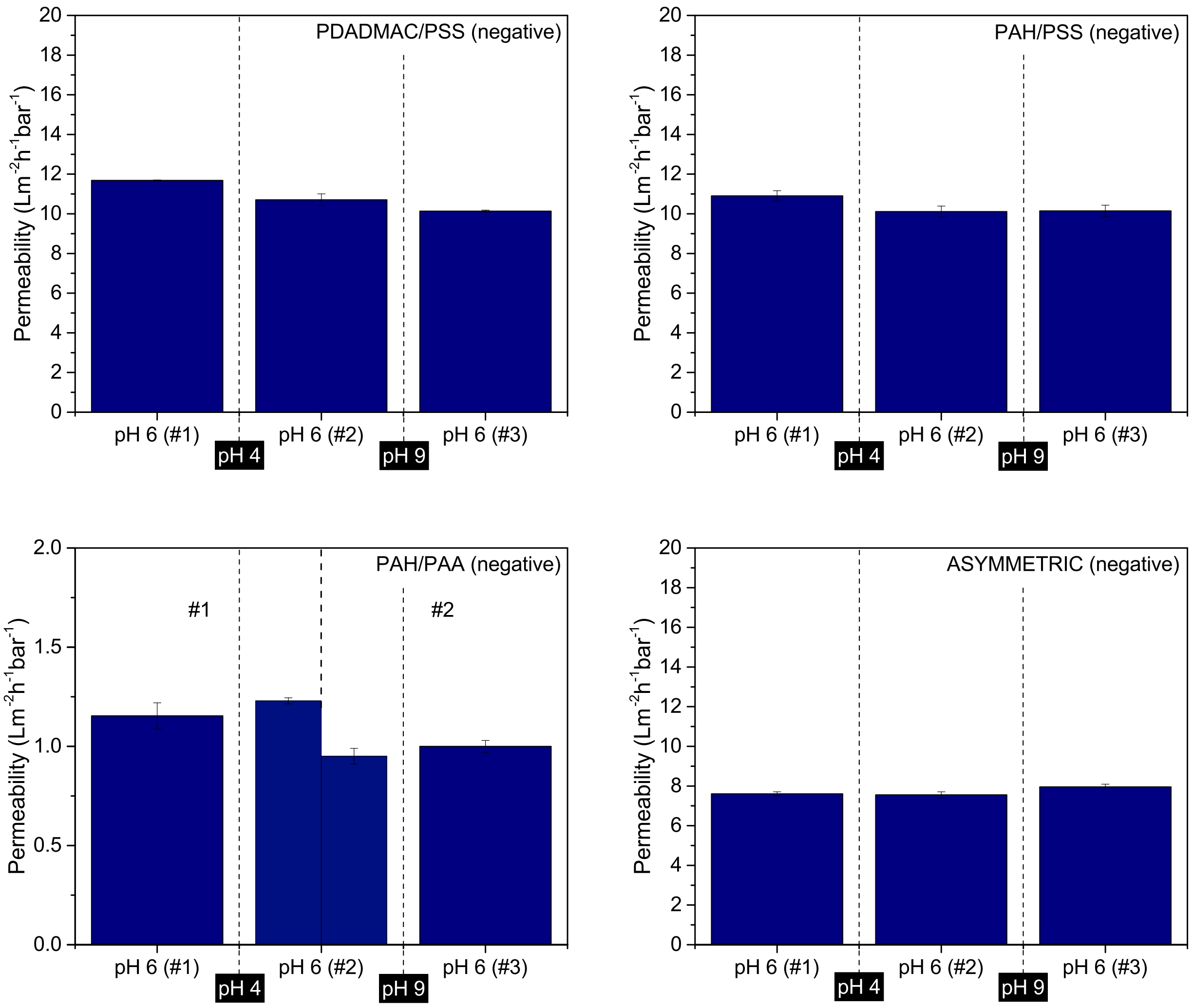

Figure S11: Reversibility behavior of negatively ending PEM membranes during pure water permeability ($Lm^{-2}h^{-1}bar^{-1}$) experiments as a function of feed solution pH (-). Mind the scale for PAH/PAA. Error bars display the standard error (sample size n=4).

Figure S12: MWCO and salt retention reversibility positive ending membranes

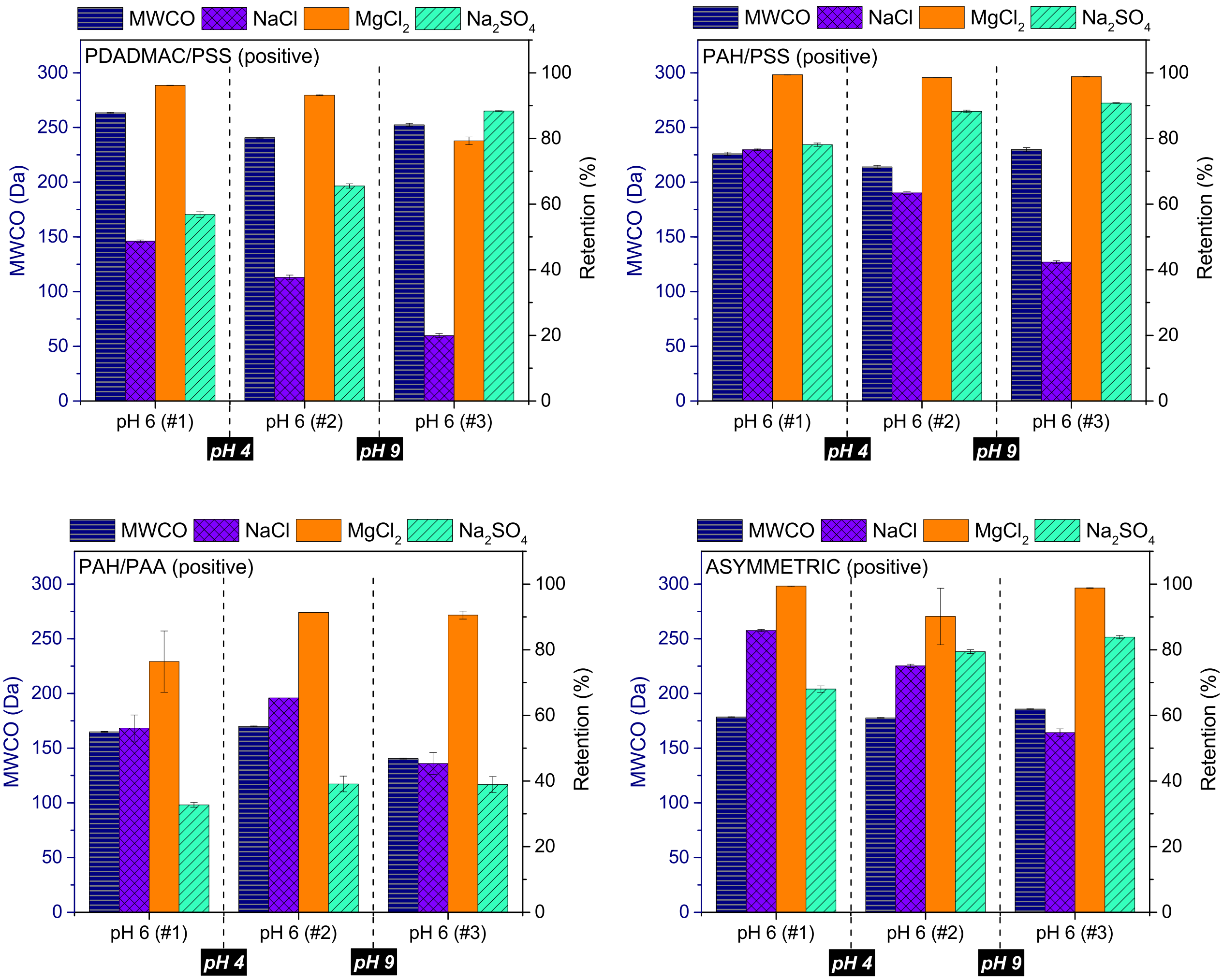

Figure S12: Reversibility behavior of positively ending PEM membranes during MWCO (Da) (**left y-axis**) and salt retention (%) (**right y-axis**) experiments as a function of pH (-). Note: PAH/PAA pH 6(#2) data displays MWCO and Na_2SO_4 reversibility from batch #2 of PAH/PAA membranes. The obtained data nicely matches with values obtained from batch #1. NaCl and $MgCl_2$ data originates from PAH/PAA batch #1. Due to broken membranes less data points were obtained, hence no error bars are shown. Error bars display the standard error (sample size n=4).
